# Role of autophagy in angiogenic potential of vascular pericytes

**DOI:** 10.3389/fcell.2024.1347857

**Published:** 2024-02-06

**Authors:** Soheil Zamen Milani, Aysa Rezabakhsh, Mohammad Karimipour, Leila Salimi, Narges Mardi, Maryam Taghavi Narmi, Fatemeh Sadeghsoltani, Ferzane Valioglu, Reza Rahbarghazi

**Affiliations:** ^1^ Student Research Committee, Tabriz University of Medical Sciences, Tabriz, Iran; ^2^ Cardiovascular Research Center, Tabriz University of Medical Sciences, Tabriz, Iran; ^3^ Stem Cell Research Center, Tabriz University of Medical Sciences, Tabriz, Iran; ^4^ Biotechnology Research Center, Tabriz University of Medical Sciences, Tabriz, Iran; ^5^ Technology Development Zones Management CO., Sakarya University, Sakarya, Türkiye; ^6^ Department of Applied Cellular Sciences, Faculty of Advanced Medical Sciences, Tabriz University of Medical Sciences, Tabriz, Iran

**Keywords:** pericytes, vascular function, autophagy, protective/detrimental activity, pathological conditions

## Abstract

The vasculature system is composed of a multiplicity of juxtaposed cells to generate a functional biological barrier between the blood and tissues. On the luminal surface of blood vessels, endothelial cells (ECs) are in close contact with circulating cells while supporting basal lamina and pericytes wrap the abluminal surface. Thus, the reciprocal interaction of pericytes with ECs is a vital element in the physiological activity of the vascular system. Several reports have indicated that the occurrence of pericyte dysfunction under ischemic and degenerative conditions results in varied micro and macro-vascular complications. Emerging evidence points to the fact that autophagy, a conserved self-digestive cell machinery, can regulate the activity of several cells like pericytes in response to various stresses and pathological conditions. Here, we aim to highlight the role of autophagic response in pericyte activity and angiogenesis potential following different pathological conditions.

## 1 Introduction

Brain and other tissue pericytes are one of the main cellular components involved in vascular integrity and the regulation of blood flow (Hattori, 2022). Pericytes are located at the abluminal surface of vascular tissue and enwrap the endothelial layer via juxtacrine interaction, namely, myoendothelial gap junction (Vicario and Parenti, 2022). The occurrence of varied pathological conditions such as ischemic stroke, infarction, and degenerative conditions can increase pericyte atresia and detach them from the endothelial layer (Uemura et al., 2020). Under pathological conditions, the release of degrading enzymes with severe vascular injury contributes to the development of hemorrhagia, and the recruitment of immune cells (Spronk et al., 2021). Besides, the loss of vascular integrity and cell-to-cell junction leads to local hematoma inside the cranial cavity. It is evident the increase in intracranial pressure promotes brain tissue injury (Wang, 2010; Yang et al., 2022). Along with these descriptions, reciprocal cross-talk between different vascular cells such as pericytes, endothelial cells (ECs), and other cells is critical in the homeostasis and physiology of blood vessels (Alarcon-Martinez et al., 2022). It has been shown that the loss of homotypic pericyte‒to‒pericyte junction, and the heterotypic interaction of pericytes with ECs, and other cells contributes to the disruption of vascular integrity (Alarcon-Martinez et al., 2022). As a common belief, pericytes can regulate the permeability of the blood-brain-barrier (BBB) interface via the production of various signaling molecules. These factors support the maintenance of tight junctions between the ECs and guide the astrocyte polarization endfeet (Geranmayeh et al., 2019a).

Emerging data have revealed the fundamental role of several signaling pathways, especially autophagy, in the function of pericytes under physiological and pathological conditions (Fu et al., 2016). Autophagy, a self-digestive and catabolic system, has a crucial role in the maintenance of cell homeostasis (Shabkhizan et al., 2023). The autophagy machinery is a scavenging system to exclude injured organelles and misfolded proteins. This system can recycle the digested substrates to compensate for the fatal energy crisis. These features make the cells resistant to several insulting conditions (Hassanpour et al., 2021; Shahabad et al., 2022). The activation of adaptive (normal) autophagic response is associated with pericyte functional characteristics and vascular homeostasis (Molina et al., 2022). It has been thought that both impaired autophagic response and excessive autophagy response not only cannot protect the host cells like pericytes after being exposed to pathological conditions but also it can accelerate cell death mechanisms (Fu et al., 2016). Here, in this review article, the role of autophagy was highlighted in angiogenic activity and function of pericyte in several pathologies. How and by which mechanisms adaptive autophagy can regulate pericyte function, increase their resistance to insulting conditions, *i.e.*, metabolic disorders and inflammatory response, and restore the cellular homeostasis is at the center of debate. Besides, the detrimental effects of excessive and impaired autophagy were also discussed in vascular pericytes. It seems that this review article can help us in the understanding of protective/detrimental role of autophagy in pericytes functional characterises under different biological situations.

## 2 Pericytes function and activity

Pericytes exhibit heterogeneous sources and originate from the mesoderm and neural crest. The cell can support the vascular integrity by wrapping the vascular endothelial layer. A fraction of pericytes within the central nervous system and lymphoid organs such as the thymus are from the neural crest origin while in the heart, lungs, liver, and gut mesothelium is the source of pericytes (Dias Moura Prazeres et al., 2017; Harrell et al., 2018). From the morphological aspect, pericytes constitute three sub-types as follows; mesh pericytes, ensheathing pericytes, and thin-strand pericytes (Brown et al., 2019). Although all pericyte types can share a protruding soma the existence of varied cellular processes with distinct morphologies helps them to function in different tissues (Attwell et al., 2016; Alarcon-Martinez et al., 2021). It is suggested that ensheathing pericytes are juxtaposed to the arteriole-capillary junction via projections wrapping the vascular structure. The mid-capillaries are the source of thin-strand pericytes with long, thin processes tracing the external vascular surface. The last cell type is mesh pericytes longitudinal short processes are located on the abluminal surface of post-capillary venules and the capillaries (Attwell et al., 2016; Berthiaume et al., 2018).

Along with morphological features, the molecular identity and genomic profile have revealed different pericyte types. Importantly, the molecular profile is not exclusive to pericytes, and the type, function, and location of these cells can affect the molecular signature (Bergers and Song, 2005). Intracellular proteins like desmin, alpha-smooth muscle actin (α-SMA), regulator of G protein signaling 5 (RGS-5), cell surface proteins like neuron-glial antigen 2 (NG2), and platelet-derived growth factor receptor beta (PDGFR-β) are the main target molecules used commonly for pericyte identification (van Splunder et al., 2023). Unfortunately, desmin belonging to type III contractile filaments can be found in different muscle cell types (Di Conza et al., 2023). Likewise, α-SMA is a cytoskeletal protein and presents in smooth-muscle cells and fibroblasts (Zheng et al., 2023). RGS-5 is a protein that activates GTPase proteins and disrupts sphingosine-1-phosphate, endothelin-1, angiotensin II, and PDGF-induced signaling in cultured cells (Bondjers et al., 2003; Cho et al., 2003). RGS-5 is an angiogenesis marker and its expression promotes neovascularization rate (Lu et al., 2023). Of Note, NG2 is a chondroitin sulfate proteoglycan expressed on the surface of pericytes during vasculogenesis and angiogenesis (Stallcup, 2002). This factor is not detectable in mature vascular, indicating the role of this factor in the induction and progression of angiogenesis (Chen et al., 2022). PDGFR-β with a tyrosine-kinase activity is crucial for the commitment of stem cells toward pericytes. Besides, the activity of this receptor in the angiogenesis process has been proved (Hellström et al., 1999; Betsholtz, 2004; Bergers and Song, 2005). It is thought that differences in origin, molecular profile, and morphology lead to unique pericyte activity (Alarcon-Martinez et al., 2021). For instance, the production of α-SMA, myosin, and tropomyosin especially in ensheathing pericytes increases the cell contractibility, resulting in the regulation of blood flow rate (Rucker et al., 2000; Alarcon-Martinez et al., 2018).

**FIGURE 1:**
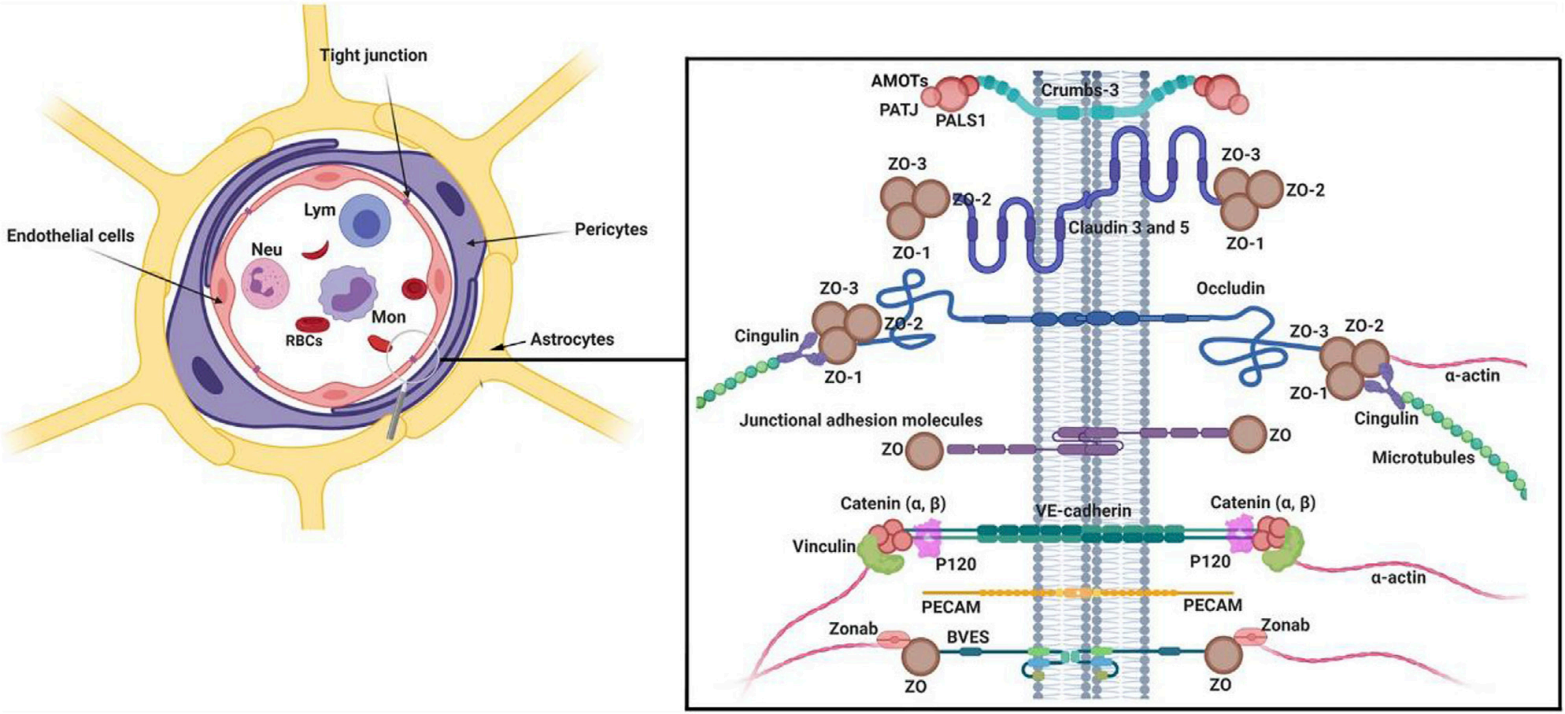
Multicellular components of BBB interface. The monolayer ECs are surrounded by pericytes and astrocyte endfeet. To provide a selective barrier, ECs are juxtaposed to each other by several junctional adhesion molecules (JAMs). Molecular investigations have revealed that Claudins, occludin, and JAMs are the most important tight junction proteins in the structure of BBB. Numerous intracellular adaptor factors such as ZO-1, -2, cingulin, Jacob, and membrane palmitoylated proteins are connected to adhesion complexes. Reproduced with permission (Heidarzadeh et al., 2021). Copyright 2021, Cell and Bioscience.

The central nervous system is exposed to several compounds in blood. To prevent the uncontrolled entry of these compounds into the brain parenchyma, pericytes constitute a selective BBB interface with the collaboration of ECs and astrocytes. It is suggested that the integrity and function of BBB are extremely associated with the normal function of pericytes (Figure 1) (Geranmayeh et al., 2019b; Brown et al., 2019). Of note, histological and molecular works have provided evidence that the pericyte/EC ratio is higher in the brain microvascular system compared to other vessel types, indicating an important role of pericytes in the BBB integrity (Uemura et al., 2020). In newly generated blood vessels, the recruitment of pericytes results in the functional integrity of the vascular barrier (Uemura et al., 2020).

The production of several signaling molecules by pericytes supports the maintenance of tight conjunction between the ECs and the attachment of astrocyte end-feet (Ma et al., 2018). Ultrastructural analyses have shown that pericytes are physically sandwiched between ECs and astrocyte endfeet (Armulik et al., 2010). To be specific, astrocytes are attached to the surface of BBB pericytes using certain channels like aquaporin 4, and Kir4.1 with a regular distribution. Loss of the pericyte layer and weakening contact between pericytes and ECs leads to the reduction of aquaporin 4, α-syntrophin, and basal membrane protein laminin in brain astrocytes (Armulik et al., 2010). Under pathological conditions, pericytes can exhibit phagocytic activity and are involved in antigen presentation to local leukocytes (Thomas, 1999; Jansson et al., 2014; Rustenhoven et al., 2017). Upon exposure to inflammatory cytokines like IL-1β and TNF-α, pericytes produce and release other inflammatory cytokines such as metalloprotease 9, leading to increased permeabilization of BBB interface and the possibility of encephalitis (Herland et al., 2016). Pericytes are tightly connected to ECs and this interaction enables pericytes to control dynamic growth, migration, and phenotype acquisition of ECs via the release of Notch3, VEGF, angiopoietin 1, PDGF-BB, etc. (Gaengel et al., 2009; Lee et al., 2010). Following the release of pro-angiogenesis factors, the physical connection of pericytes to the vascular surface is loosened and thus ECs enter the proliferation state (Yancopoulos et al., 2000; Bergers and Benjamin, 2003). While pericytes are recruited to the abluminal surface of vessels during the maturation of vessel structure. The production of specific factors such as angiopoietin 1, and PDGF-BBB increases the physical contact of pericytes around vessels (Ribatti et al., 2011).

## 3 Autophagy mechanisms and molecular machinery

**FIGURE 2:**
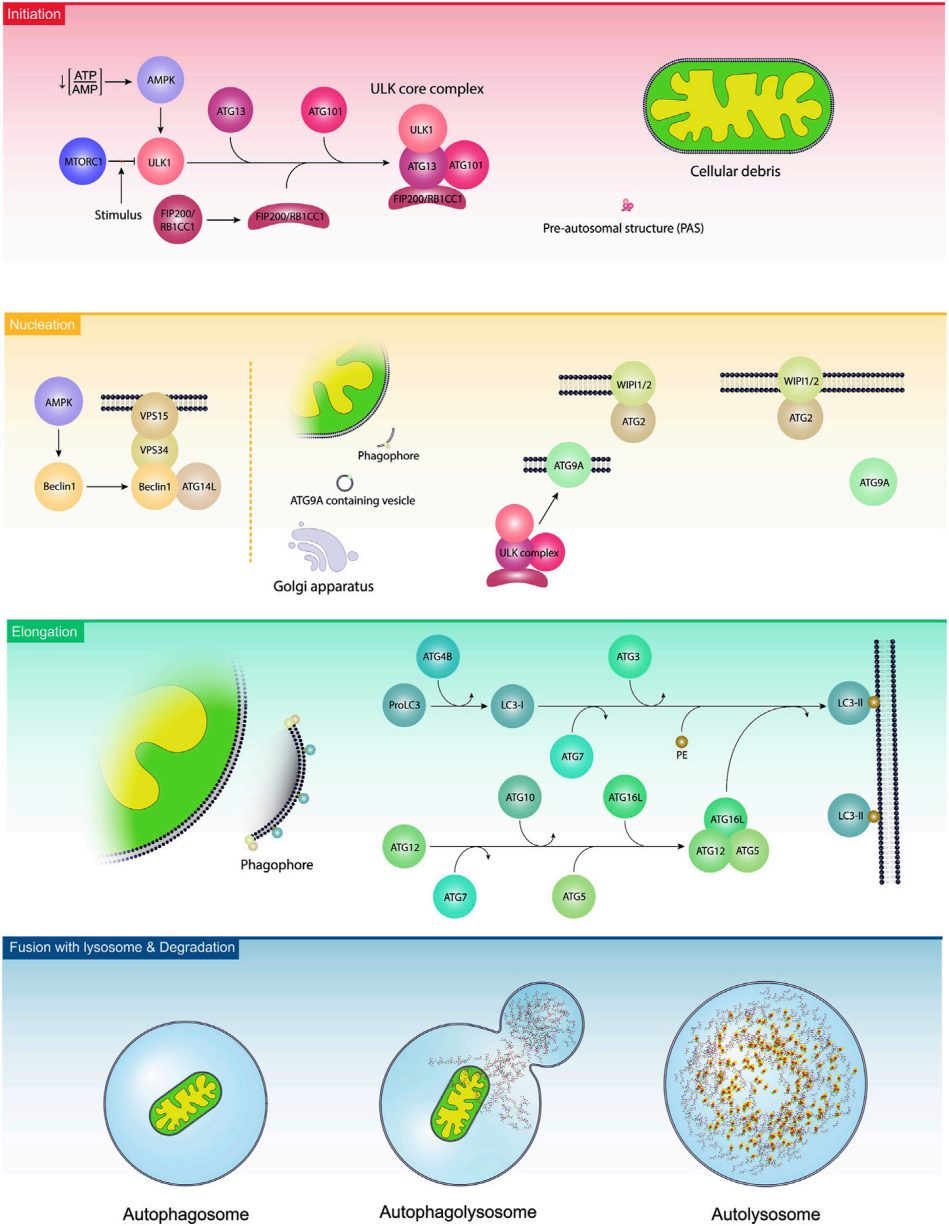
Autophagy activation steps. In the initiation step, the ULK1 complex is stimulated due to the existence of several stimuli, leading to the reduction of ATP/AMP ratio, activation of AMPK, and inhibition of mTORC1. Along with the activation of the ULK1 complex, ATG13, ATG101, and FIP200/RB1CC1 were also engaged and are localized to pre-autosomal structures. The procedure is continued with the nucleation step, leading to the generation of phagophores. In this step, namely, elongation, BCLIN1 activates other complexes like ATGL14, VPS34, and VPS15. In the elongation step, ATGs and lipids are added to the phagophores. Finally, phagophores generate autophagosomes and fuse with the lysosomes to form autophagolysosomes.

Autophagy is a cellular process to exclude defective organelles and misfolded proteins under normal and pathological conditions (Rezabakhsh et al., 2018). In a broad classification, autophagy is detected in three distinct forms macroautophagy, microautophagy, and chaperone-mediated autophagy (CMA) (van Splunder et al., 2023). Macroautophagy, herein referred to as autophagy, is the dominant autophagy form in eukaryotic cells. In constitute and inducible macroautophagy, injured compounds are sequestrated into bilayer membrane vesicles, namely, autophagosomes. Further fusion of autophagosomes with lysosomes leads to the formation of autophagolysosomes and the degradation of cellular materials (Li et al., 2023). The term microphagy refers to the degradation of several substances via invagination into the lysosomal lumen (Wang et al., 2023). The latter autophagy type, CMA, is a selective autophagy that targets proteins based on specific motifs and is involved in supplying amino acids after degradation and recycling procedures (Tasset and Cuervo, 2016). It is believed that either selective or non-selective autophagy can control cell activity by targeting certain intracellular entities (Liu et al., 2020). Selective autophagy such as mitophagy (autophagy of mitochondria), reticulophagy (autophagy of endoplasmic reticulum), nucleophagy (autophagy of the nucleus), ribophagy (autophagy of ribosomes), lipophagy (autophagy of lipids), RNautophagy and DNatophagy, xenophagy (autophagy of invading bacteria of viruses), and autophagy of specific proteins such as ferritinophagy are as examples (Ichimiya et al., 2020; Liu et al., 2020). From the molecular aspect, the formation of autophagosome is controlled in three distinct steps as follows; initiation, nucleation, and elongation (Ichimiya et al., 2020). Following the induction of autophagy response in host cells, pre-autophagosomal structures (PAS) are generated. Along with these changes, the ULK-ATG13-ATG101-FIP200 complex, an autophagy initiation molecular assembly, recruits autophagy-related proteins (ATG) to advance the formation of the phagophores, and thus autophagosomes (Figure 2) (Mizushima, 2010). Molecular studies have revealed that several effectors such as the mechanistic target of rapamycin kinase complex 1 (mTORC1) and AMP-activated protein kinase (AMPK) can regulate the autophagic response inside the cells (Fritzen et al., 2016; Losier et al., 2019). It was suggested that mTORC1 binds to ULK1, an enzyme with N-terminal kinase activity, and inhibits its activity. Upon the reduction of the ATP/AMP ratio, the activation of AMPK recruits ULK1 and BECN1, resulting in the stimulation autophagy signaling pathway (Bujak et al., 2015; Klionsky et al., 2021). BECN1 is a scaffolding protein that facilitates the formation of class III PI3K complex (Ye et al., 2023). In the class III PI3K complex, the direct interaction of ATG14L and UVRAG with BECN1 leads to the activation of VPS34, and VPS15 (Ye et al., 2023). It is thought that VPS34 is a catalytic PI3K and participates in the formation of PI3-phosphate (PI3P). Based on molecular investigations, PI3P is a prerequisite for the formation of autophagosomes and the recruitment of WIPI to the bilayer membrane (Noda et al., 2010). With the arrival of the FIP200/RB1CC1 complex to the PAS, these structures are elongated to acquire a crescent shape (Suzuki et al., 2007; Cao et al., 2021). Along with these changes, the phosphorylation of ATG13 and its binding to ULK1 generates a ULK-ATG13 dimer, leading to the stimulation of autophagosomes (Wong et al., 2013). In the latter steps, the attachment of the ULK-ATG13 dimer to FIP200 and ATG101 leads to the formation of the ULK core complex (Li et al., 2020). These changes promote the formation of class III PI3K complex and ATG9A system (Li et al., 2020). Emerging data have shown that ATG9A is involved in the transfer of lipid droplets to autophagosomes, and the generation of ATG9A-loaded vesicles (Mailler et al., 2021). The enzymatic activity of the ULK complex leads to the phosphorylation of ATG9A which is essential to recall WIPI1/2 and LC3 to the PAS site (Papinski et al., 2014). With the fusion of ATG9A-containing vesicles with PAS, ATG9A molecules are released and recycled to the newly generated vesicles (Yamamoto et al., 2012). This phenomenon is tightly controlled via the activity of the WIPI1/2-ATG2 complex (Suzuki et al., 2007; Polson et al., 2010). The WIPI1/2-ATG2 complex is juxtaposed to the expanding edges of the vesicle membrane and regulates the closure and expansion of these edges (Li et al., 2020). The elongation of phagophores is promoted by the addition of two ubiquitin-like conjugation systems ATG12 and ATG8 (LC3) conjugation systems. The physical contact of ATG12 with ATG5 leads to the formation of irreversible conjugate and lipidation of LC3 (Noda, 2023). To activate ATG12, the function of an E1-like enzyme ATG7 is required. Upon activation, ATG12 attaches to ATG5 by an E2-like enzyme ATG10. This complex is stabilized after the addition of ATG16L. The ATG12-ATG5-ATG16L complex is a stimulatory signal for the activation of the LC3 conjugation system (Xiang et al., 2023). Inside the cells, LC3 is initially synthesized as proLC3 (Yang et al., 2015), and its C-terminal is cleaved by ATG4B to form LC3-I with an exposed C-terminal glycine. Subsequently, LC3-I is activated by ATG7 and conjugated to the amino group of phosphatidylethanolamine (PE) by ATG3, an E2-like enzyme. The ATG12-ATG5-ATG16L complex acts as an E3 enzyme for the conjugation reaction to form LC3-II, a membrane-bound protein (Amirian et al., 2023). LC3-II is involved in the fusion of membranes and the selection of the autophagy targets (Kirkin et al., 2009). The elevation in LC3-II levels during autophagy makes it the desirable biomarker for monitoring autophagy response (Hussain et al., 2023). The autophagosomes mature after fusion with the lysosomes by recalling several Rab proteins (mainly Rab7), and SNARE complex. The LC3 decorated autophagosomes are physically attached to RAB7 and this interaction facilitates the formation of autophagolysosomes (Zhao et al., 2021). After degradation of cargo, the breakdown components are directly released into the extracellular matrix or returned into the cytosol for anabolic purposes (Shim et al., 2021).

## 4 Role of autophagy in pericyte activity

Whether and how autophagy mechanisms can regulate the dynamic activity of pericytes within the vascular structure has been at the center of the debate. Emerging data have indicated that autophagy is closely associated with the function of pericytes in several vessel types (Fu et al., 2016). As above-mentioned, pericyte homeostasis is essential and data have reported both autophagy deficiency and overactivity can lead to pericyte dysfunction under several pathological conditions (Fu et al., 2016).

### 4.1 Diabetic conditions

Previous data have confirmed the inevitable role of autophagy on bovine retinal pericyte migration in *in vitro* conditions (Lin et al., 2022). The exposure of bovine retinal pericytes to advanced glycation end products (AGEs) led to autophagy activity (LC3-II/LC3-I ratio↑, and P62↓) and enhanced migration properties without changes in the survival rate. In retinal pericytes, the production of metalloproteinase-2 (MMP-2) and phosphorylation of focal adhesion kinase (FAK) were elevated (Lin et al., 2022). Of note, incubation of these cells with chloroquine, an autophagy inhibitor, can blunt the increase of FAK and MMP-2. It was suggested that these effects could be related to the activation of AMPK and the interaction of NBR1 [an autophagy cargo receptor] with FAK. It also raised the possibility of autophagosome attachment to the focal adhesions in the cell periphery (Kenific and Debnath, 2016). Direct evidence for the reduction of focal adhesion stability in pericytes exists when autophagic NBR1 is induced in an AMPK-dependent manner. These apparent morphological changes can increase the leakage of BBB and retinal vessels under diabetic conditions (Lin et al., 2022). Owing to its protective properties, autophagy can protect different cell types after exposure to several insults, however, it has been proposed that the over-activation of autophagic machinery can mediate cell dysfunction and kill the cells in severe situations (Rezabakhsh et al., 2017). In this scenario, the incubation of human retinal pericytes with higher doses of oxidized LDL (100–200 mg/L) stimulated excessive autophagic and triggered apoptotic cell death while the lower concentration of oxidized LDL (25–200 mg/L) can lead to reticulum endoplasmic stress and activation of autophagy in a JNK-dependent manner without inducing apoptosis (Fu et al., 2016). These data support the notion that the interplay between autophagy and other signaling pathways is critical in cell survival–death balance. To be specific, under relatively mild stress situations different cells and pericytes can apply autophagic response as a cytoprotective tool but extreme stresses beyond certain thresholds can lead to the activation of cell-death signaling pathways like apoptosis. Of course, it should not be forgotten that there is a close cross-talk between autophagy and other signaling pathways, like the Wnt molecular cascade (Lorzadeh et al., 2021). Data have confirmed that the status, stimulation, and/or inhibition, of other signaling cascades can alter the function of autophagy and vice versa (Lorzadeh et al., 2021). For instance, the modulation of several Wnt effectors can affect the autophagy response under stressful conditions. Wnt3a, a Wnt ligand, can regulate the activity of AMPK and thus autophagy status inside the target cells (Ríos et al., 2018). In an experiment, Ye and co-workers indicated that diabetic retinopathy is associated with a close interaction between autophagy and the Wnt signaling pathway (Ye et al., 2021). They found that the inhibition of autophagy response led to the downregulation of the Wnt signaling pathway and thus an impaired angiogenesis activity. In diabetic *db/db* mice, the physiological activity of autophagy can alleviate the detrimental effects of metabolic abnormalities of course when the relevant molecular machine works properly. Despite the stimulation of BCLN1 and LC3 in vascular cells and retinal cells after being exposed to prolonged diabetic conditions, P62 activity is downregulated, leading to an incomplete autophagic response and the accumulation of exhaust materials inside the cells (Ye et al., 2021). That said, the accumulation of P62 at later autophagic steps not only promotes cell injury but also can abolish functional behavior of progenitor cells and even differentiation toward vascular cells (Rezabakhsh et al., 2017).

**FIGURE 3:**
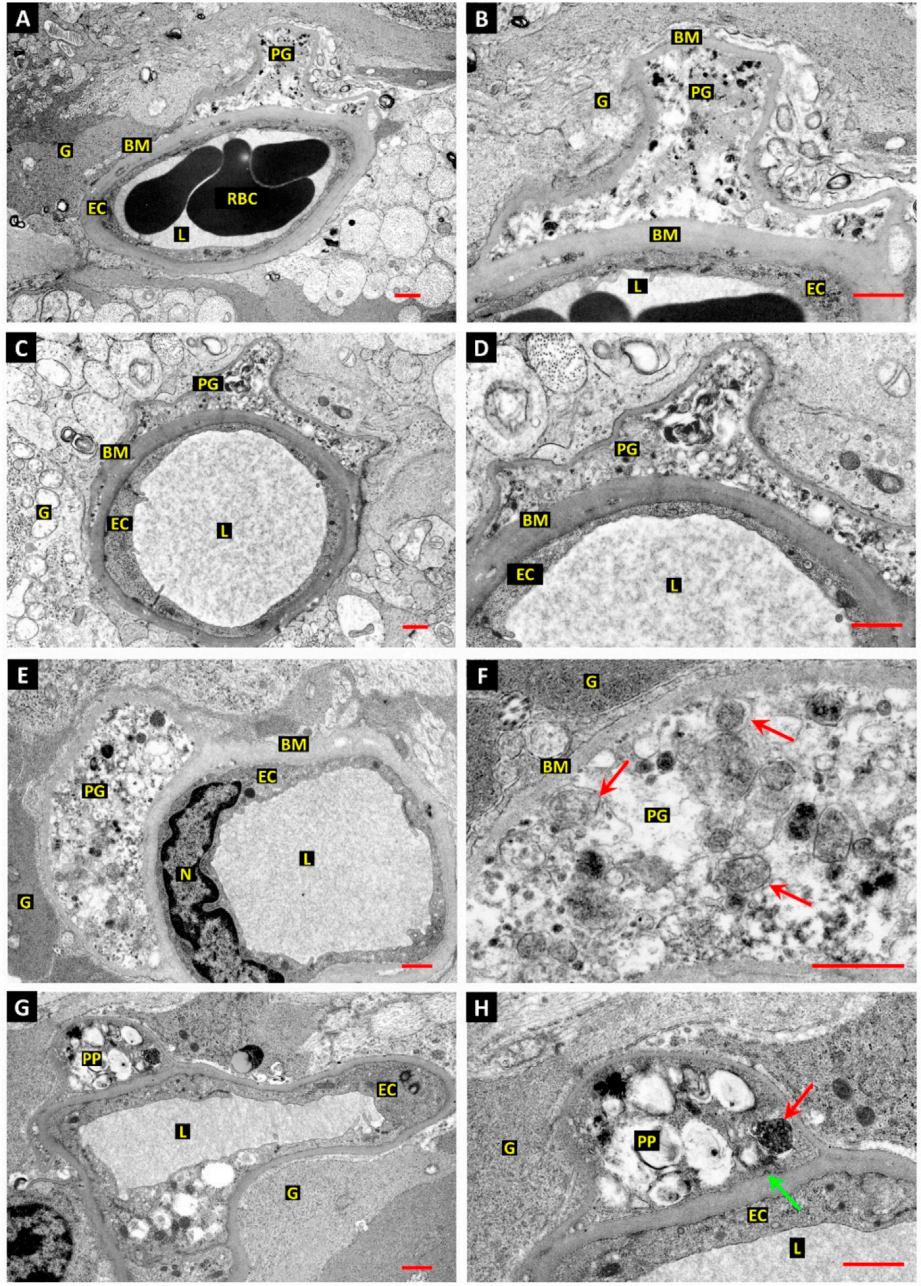
The relationship between autophagic response and retinal pericyte death in a dog with diabetes mellitus **(A–H)**. Ultrastructural images revealed dilated capillaries in the retina with normal ECs and a lack of enwrapping pericytes. Along with basal membrane thickening, numerous vesicles containing electron-dense substrates are accumulated inside the pericyte cytoplasm **(E,F)**. Vacuoles with heterogeneous materials are seen in the pericyte projections, indicating autophagy stimulation [red arrows; **(F)**]. Dense and dark vacuoles (red arrow) are autophagosomes. The green arrow indicates a clathrin-coated pit at the inner surface of the basal membrane. Scale bar: 1.0 µm; Pericyte process: PP; Red blood cells: RBC; Vessel lumen: L; Glial processes: G; Basal membrane: BM; Pericyte ghost: PG; and Endothelial cell nucleus: N. Reproduced with permission (Gardiner and Stitt, 2022a). Copyright 2022, International Journal of Translational Medicine.

Given the intricate and complex association of metabolic disorders with the vascular unit, it is postulated that a diabetic condition is likely to alter the function of cells in the BBB interface integrity (Li et al., 2022). Lee and co-workers found that cellular components can affect the integrity of BBB via the interchange of metabolic byproducts (Lee et al., 2022). ECs uptake glucose via glucose transporter 1 (GLUT1) and produce considerable levels of lactate by glycolysis pathway which is further taken up by juxtaposed pericytes. The internalized lactate is involved in the regulation of energy metabolism and biosynthetic processes (Lee et al., 2022). Interestingly, glycolysis is the main energy-producing mechanism in ECs in which about 90% of intracellular glucose can be converted into lactate via glycolysis (Faulkner et al., 2020; Lee et al., 2022). Diabetic conditions can activate endothelial GLUT1 to internalize glucose and trigger the glycolysis process and mTORC1 function in the early stages. However, prolonged hyperglycemic status dysregulates the glucose uptake and autophagy response in BBB ECs, resulting in vascular cell injuries (Madrakhimov et al., 2021). In another study, ultrastructural analyses have revealed the formation of autophagic vacuoles (autophagosomes) in retinal vessel mural cells and pericytes of diabetic patients and dogs with a subsequent fusion of lysosomes while a low number of autophagosomes were detected in ECs (Figure 3) (Gardiner and Stitt, 2022a). One reason would be that ECs are front-line cells and are directly exposed to diabetic serum and toxic byproducts. This may lead to progressive EC death and possibly inhibition of protective autophagy response at early stages compared to the pericytes and mural cells located at the abluminal side (outer layers) of the vascular structure. Another reason for the accumulation of autophagosomes in diabetic pericytes is that cells are more sensitive to glucose fluctuation and exhibit more vulnerability to apoptotic conditions rather than endothelial lineage (Ghanian et al., 2018). Irrespective of the protective and harmful roles of autophagy on pericytes and mural cells within the retinal capillaries, it was suggested that prolonged diabetic conditions can also affect cells at the outer layers. As such, hyperglycemia facilitates the efferocytosis of apoptotic pericytes with excessive autophagy activity by recalling juxtavascular microglia after the stimulation of the CX3CR1 signaling pathway (Blume et al., 2020; Gardiner and Stitt, 2022b). It was suggested that retinal pericytes fundamentally differ from the adjacent ECs in terms of mitochondrial activity and intracellular reactive oxygen species (ROS) levels. Upon exposure to hyperglycemic conditions, retinal pericytes tend to use an oxidative phosphorylation system for the production of ATP as the main power supply, increasing intracellular ROS contents (Ghanian et al., 2018). There are several proposed mechanisms associated with the reduction of autophagolysosome formation and subsequent cell injuries in diabetic patients (Zheng et al., 2020).

Recent works have established that the diabetic milieu contributes to lysosomal malfunction which in turn increases the possibility of autophagic stress and stagnation instead of adaptive autophagy (Zheng et al., 2020). It is thought that in diabetic cells the maturation of lysosomal Cathepsin D and thus the enzymatic activity of lysosomes are reduced. Concurrently, the hyperactivity of mTORC1 *per se* affects lysosomal maturation and activity via the promotion of phosphorylated ULK1 and reduction of TFEB nuclear translocation following interaction with Smad3 (Kang et al., 2019; Zhang et al., 2019). Along with these changes, modulation of Akt and AMPK activity leads to the phosphorylation of the MiT/TFEs factor that limits the subcellular location of lysosomes and degradation reactions (Asrani et al., 2019). Importantly, AGE overload, as a diabetes byproduct, can downregulate the transcription of Lamp1 and intensify insufficient lysosomal degradation (Zheng et al., 2020). The alteration of fatty acid metabolism and physical attachment of AGEs to an anti-AGE receptor can increase lysosomal membrane permeability, reduction of lumen acidity, and accumulation of injured cargo inside the cells (Zhang et al., 2019). Taken together, abnormal autophagosome-lysosome fusion even after stimulation of supportive autophagy is associated with autophagic stress and cell death under diabetic conditions.

### 4.2 Autophagy and inflammation

The occurrence of ischemic stroke is associated with, inflammation, pericyte loss and BBB disintegrity (Zhang et al., 2020). Despite the direct effect of pathological conditions on BBB multi-cellular components, it is postulated that the secretome of glial cells can also affect the function of pericytes under hypoxic conditions (Geranmayeh and Rahbarghazi, 2022). The activation of autophagic response in glucose and oxygen-deprived microglial cells using metformin led to phenotype shifting into the M2 type (Geranmayeh and Rahbarghazi, 2022). Co-culture of pericytes with metformin-treated microglia can increase pericyte maturation under hypoxic and hypoglycemic conditions. Under such conditions, Sox2/NG2 ratio is increased (Geranmayeh and Rahbarghazi, 2022). It was suggested that NG2-expressing pericytes can make physical contact with ECs via the activation of the integrin signaling pathway (Stallcup, 2018). These data indicate that autophagy can affect the differentiation potential of pericytes under inflammatory conditions. Yet, how autophagy is involved in the orientation of progenitors toward functional pericytes remains to be determined and needs further investigation. The participation of autophagy machinery in the differentiation of CD146^+^ pericyte progenitors toward mature pericytes and ECs was further confirmed by Hassanpour and co-workers (Hassanpour et al., 2020). They found that the stimulation of autophagy using metformin increased lineage-dependent proteins along with the alteration of autophagy markers while inhibition of autophagy using hydroxychloroquine blunted these effects via the increase of pro-inflammatory cytokines like IL-6 and TNF-α (Hassanpour et al., 2020). These features show that the activation of autophagy would occur in response to insulting conditions to reduce the levels of inflammatory cytokines. Whether inflammatory cytokines can alter the autophagic response in brain pericytes under pathological conditions should be addressed. In this regard, Julie and co-workers showed that the exposure of mouse BBB pericytes to IL-1β did not alter the expression of autophagy factors (BCLN1, LC3, and P62/SQSTM1), mTOR, and autophagic flow (Julie et al., 2022). On the contrary, Sil and colleagues found that the induction of pro-inflammatory conditions using psychostimulants such as cocaine can stimulate BBB pericyte dysfunction via the alteration of autophagy (Sil et al., 2019). Data indicated that cocaine can increase TNF-α, IL-1β, and IL-6 levels, autophagosome number, ER stress pathways, and autophagy markers in a time-dependent manner. It is postulated that the production of pro-inflammatory cytokines can initiate autophagy response, however, impaired autophagy machinery contributes to pericyte dysfunction under inflammatory conditions (Sil et al., 2019). The pioneer works have also indicated that excessive production of inflammatory cytokines like IL-1β during Alzheimer’s disease can promote harmful autophagy within the brain parenchyma (Álvarez-Arellano et al., 2018).

**FIGURE 4:**
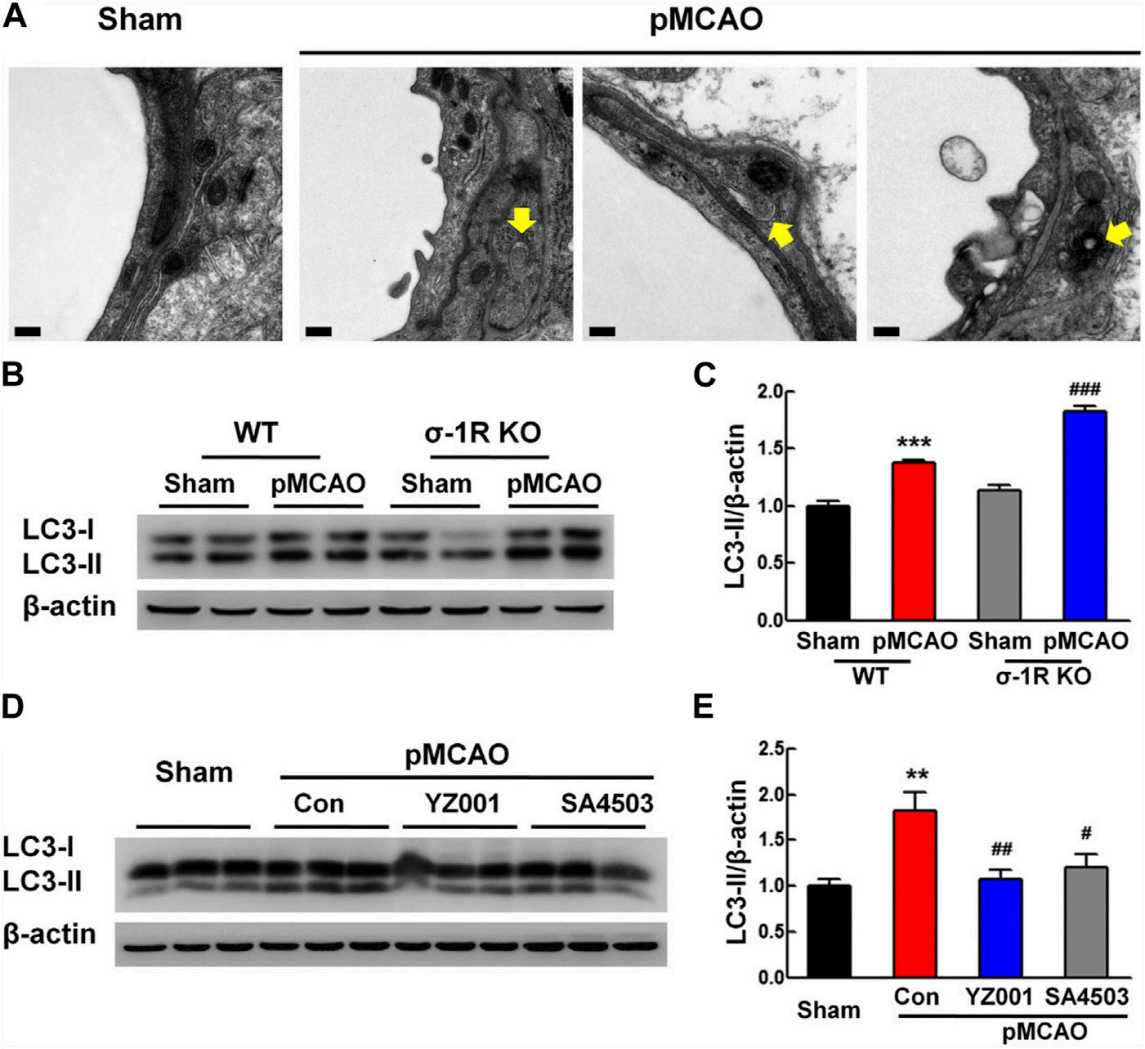
Effect of σ-1R agonist, YZ001, on the autophagic activity of pericyte in σ-1R knock-out mice subjected to photothrombotic middle cerebral artery occlusion **(A–E)**. TEM images revealed the existence of autolysosomes and autophagosomes (yellow arrows) in pericytes 24 h after induction of ischemic conditions (n = 4; Scale bar = 0.2 μm). The suppression of σ-1R leads to stress autophagy in the ischemic group [**(B,C)**; n = 3]. ***p < 0.001 versus the wild-type sham group and ^###^p < 0.001 versus the wild-type ischemic group. LC3-II/LC3-I ratio and total LC3 content were increased in σ-1R knock-out mice compared to the control group. Application of σ-1R agonist, YZ001, alleviated ipsilaterally the overactivity of autophagy in ischemic mice [**(D,E)**; n = 3]. ***p < 0.001 versus the sham group, ^###^ *p* < 0.001 versus the ischemic-treated control group. One-way ANOVA followed by the Holm–Sidak test. Photothrombotic middle cerebral artery occlusion; pMCAO. Reproduced with permission (Zhang et al., 2020). Copyright 2020, Translational Stroke Research.

In an experiment conducted by Zhang and co-workers, they found that sigma-1 (σ-1R) receptor activity can reduce pericyte dysfunction under ischemic stroke in the brain parenchyma (Zhang et al., 2020). The induction of experimental stroke in σ-1R knock-out mice led to massive pericyte apoptosis compared to the wild-type group (Zhang et al., 2020). Besides, it is postulated that the suppression of these receptors can exacerbate the protein levels of tight junction proteins like occludin, claudin 5, and ZO-1 in BBB ECs (Zhang et al., 2020). σ-1R is an endoplasmic membrane receptor and participates in intracellular calcium homeostasis via the regulation of voltage-gated and non-voltage-gated ion channels (Soriani and Kourrich, 2019). Data indicated that stimulation of σ-1R with an appropriate agonist YZ001 increases protein levels of pericyte NG2 and thus reduces pericyte loss within the brain parenchyma (Zhang et al., 2020). Besides, σ-1R stimulation leads to reduced apoptotic changes in BBB pericytes via reduction of the Bax/Bcl-xl ratio and deactivation of Caspase-3 (Zhang et al., 2020). Along with these changes, the excessive autophagic response was modulated and reached near-to-normal levels by regulating LC3 levels (Figure 4). These apparent correlations indicate the close interplay between autophagy and apoptosis in the functional characteristic of pericytes under several pathologies. Uncontrolled and excessive autophagic response can stimulate apoptotic effectors. For instance, the overactivation of certain ATGs, especially ATG5 and 12, recall caspases via the modulation of mitochondrial function (Xi et al., 2022). Under metabolic disorders such as hyperglycemic conditions, the accumulation of AGE, and ROS increases the possibility of autophagy inhibition and apoptotic cell death (Shi et al., 2015). Of note, the existence of sulfhydryl groups in the structure of ATG3, 7, and 10 makes them sensitive to ROS oxidation (Filomeni et al., 2010). Because apoptosis is the dominant cell death mechanism in vascular cells, especially pericytes, in metabolic conditions and traumatic injury thus one can hypothesize that autophagy is inhibited under such conditions, or its overactivation provokes the apoptosis signaling pathway (Kim et al., 2023).

During several degenerative diseases, the accumulation of inclusion bodies in vascular cells increases the possibility of microvessel-associated injuries (Schultz et al., 2017). For instance, pancreatic amylin hormone can accumulate in oligomer and fibril forms inside the brain pericytes in Alzheimer’s disease (Schultz et al., 2017). Of note, the increase of the fibril form of amylin led to the reduction of NG2 levels and activity of Caspases 3 and 7. Imaging data indicated the elevation of fluorochrome-tagged LC3 in amylin-exposed pericytes, resulting in the interruption of fusion between autophagosomes and lysosomes (Schultz et al., 2017). The formation of enlarged lysosomes is associated with lysosome dysfunction. Of note, the etiologies related to the cell digestive system like lysosomes can exacerbate pathological changes via the inhibition of autophagic response in the last steps. In an experiment, an inevitable role of oxidative stress and autophagy activity was studied in brain pericytes. In this regard et al. found that the expression of TRPM2, a Ca^2+^‒permeable cationic channel, is associated with the reduction of LC3 content, reticulum endoplasmic stress, and pericyte injury after exposure to zinc oxide nanoparticles (Jiang et al., 2017). Taken together, the occurrence of inflammatory diseases and the production of specific factors can affect the autophagic flux in pericytes and reduce their resistance to cell injury.

### 4.3 Autophagy and juxtacrine (physical) activity

**FIGURE 5:**
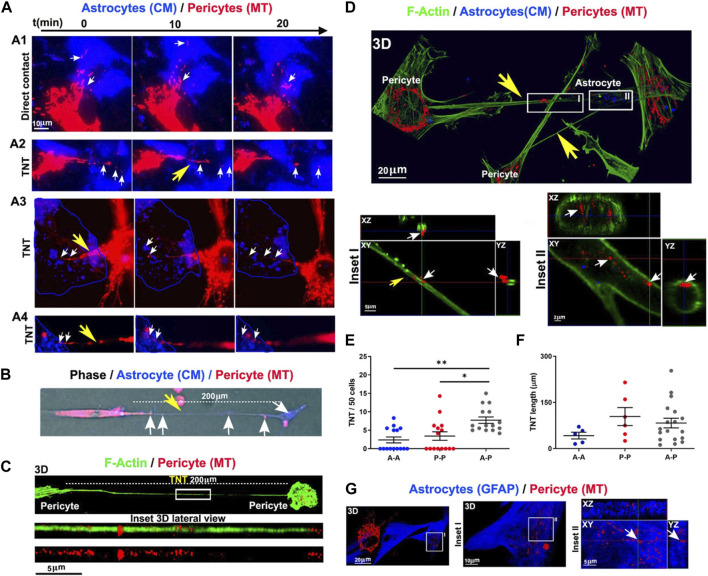
Monitoring inter pericyte-astrocyte mitochondrial trafficking *in vitro* **(A–G)**. Pericytes and astrocytes were stained with MitoTracker Deep Red (MT) and CellMask Orange (CM) and co-cultured *in vitro*. Pericytes extended projections toward the astrocytes in the co-culture system **(A)**. Epi-fluorescence imaging of TNT (200 μm in length; yellow arrow) formed between the red-colored pericyte and blue-colored astrocyte. Mitochondria are present inside the TNT (white arrows) **(B)**. Monitoring the formation of TNT between pericytes after 24 h using confocal images **(C)**. F-actin was stained with fluorescent phalloidin (green). Numerous mitochondria are visible inside the TNT (length >200 μm). Imaging of inter pericyte-astrocyte TNT formation **(D)**. TNT F-actin was stained with green fluorescent phalloidin extended from pericytes toward astrocytes with numerous mitochondria (red particles). Inside the astrocyte cytoplasm, donated mitochondria are visible (n = 3; **(D)**. Measuring homotypic, heterotypic TNT formation between pericytes (P-P), astrocytes (A-A), and pericytes with astrocytes (A-P) after 24 h *in vitro* (TNT number/field of 50 cells) [n = 3; **(E)**]. Homotypic and heterotypic-TNT length after 24 h of co-culture [n = 3; **(F)**]. Pericyte mitochondria are inside the GFAP-positive astrocytes [n = 3; **(G)**]. *p < 0.05; **p < 0.005 with Kruskal–Wallis/Dunn’s tests. Reproduced with permission (Pisani et al., 2022), Copyright 2022, Cell Death and Disease.

It has been shown that the exchange of intracellular organelles and small-sized vesicles occurs via juxtacrine activity and intercellular bridges between the multiplicity of cells within the BBB (Pisani et al., 2022). Tunneling nanotubes (TNTs) are cellular protrusions with the F-actin membrane that participate in the homotypic and heterotypic cell-to-cell connections (Pisani et al., 2022). Based on previous data, PDHA1^+^ mitochondria and LC3B^+^ autophagosomes are also present in TNTs where they can be reciprocally transferred between the cells (de Rooij et al., 2017; Pisani et al., 2022). How and by which mechanisms autophagy can regulate the activity of TNT formation in pericytes remains unknown. BBB ECs and especially pericytes form heterotypic TNTs to donate mitochondria and cease the apoptotic changes in reactive astrocytes after being exposed to oxygen-glucose deprivation in *in vitro* conditions (Figure 5) (Pisani et al., 2022). These features indicate that pericytes play a central role in the maintenance of BBB integrity by donation via nano-sized TNT structures.

Based on astrocyte activity in response to pathological conditions, excessive oxidative stress can contribute to the promotion of several proteases in the mitochondrial matrix and mitophagy response (Weidling and Swerdlow, 2019). It is postulated that the mitochondrial donation by pericytes to juxtaposed acceptor cells like astrocytes occurs under pathological conditions to regulate redox homeostasis, compensate for the lack of sufficient ATP, and reduce injuries related to deficient mitochondria (Rowe, 2020; Salmina et al., 2021; Pisani et al., 2022). After pericyte injury, the transfer of mitochondria or related particles happens from pericytes to astrocytes to reduce the probability of mitophagic death in stressed pericytes in conditions where the fragments of mitochondria are not completely lysed and recycled by lysosomes. In line with these statements, astrocytes are potent glial cells to take injured mitochondria from injured neurons for disposal and recycling, resulting in neuronal resistance and viability under ischemic conditions (Hayakawa et al., 2016; Liu et al., 2021). Whether a bidirectional transfer exists between the pericytes and juxtaposed astrocytes endfeet under physiological and pathological conditions should be answered.

Under diabetic conditions, the generation of TNTs is a compensatory mechanism to alleviate impaired autophagic response due to AGE-induced lysosomal dysfunction (Barutta et al., 2022). To be specific, the deletion of *tnfaip2*, involved in TNT formation, can affect the detrimental effects of AGE-induced autophagy and lysosome dysfunction in diabetic podocytes (Barutta et al., 2022). Alarcon-Martinez and co-workers identified vesicular transfer via TNTs in the homotypic pericyte-to-pericyte and heterotypic pericyte-to-EC connection (Alarcon-Martinez et al., 2020). Ultrastructural analyses revealed that these TNTs are extended from proximal pericyte soma to distal pericytes (Alarcon-Martinez et al., 2020). Interpericyte TNTs are involved in the regulation of capillary diameter and blood supplementation (Alarcon-Martinez et al., 2022). In glaucomatous eyes, the regulation of intra-pericyte calcium homeostasis restored the function of these cells and vascular integrity (Alarcon-Martinez et al., 2022). Without exaggeration, TNTs can be an alternative route for the exclusion of misfolded proteins and exhaust compounds via the autophagosomes where pathological conditions blunt the autophagosome-lysosome fusion. These features can in turn affect the pericyte cytotoxicity in response to autophagic stress. Using immunofluorescence imaging, it was shown that the mean diameter of inter-pericyte and pericyte-to-EC TNTs reach about 0.5 µm while the mean diameter size of autophagosome is at the range of 0.5–1.5 μm in higher creatures (Shibutani and Yoshimori, 2014). This indicates that the net transfer for large sized autophagosomes is limited compared to the small-sized counterparts. If so, large-sized autophagosomes should undergo morphological adaptation and elongation during the intercellular transfer via TNTs. The significance of TNTs in autophagosome transfer under pathological conditions should be addressed by further studies.

### 4.4 Autophagy and paracrine activity

**FIGURE 6:**
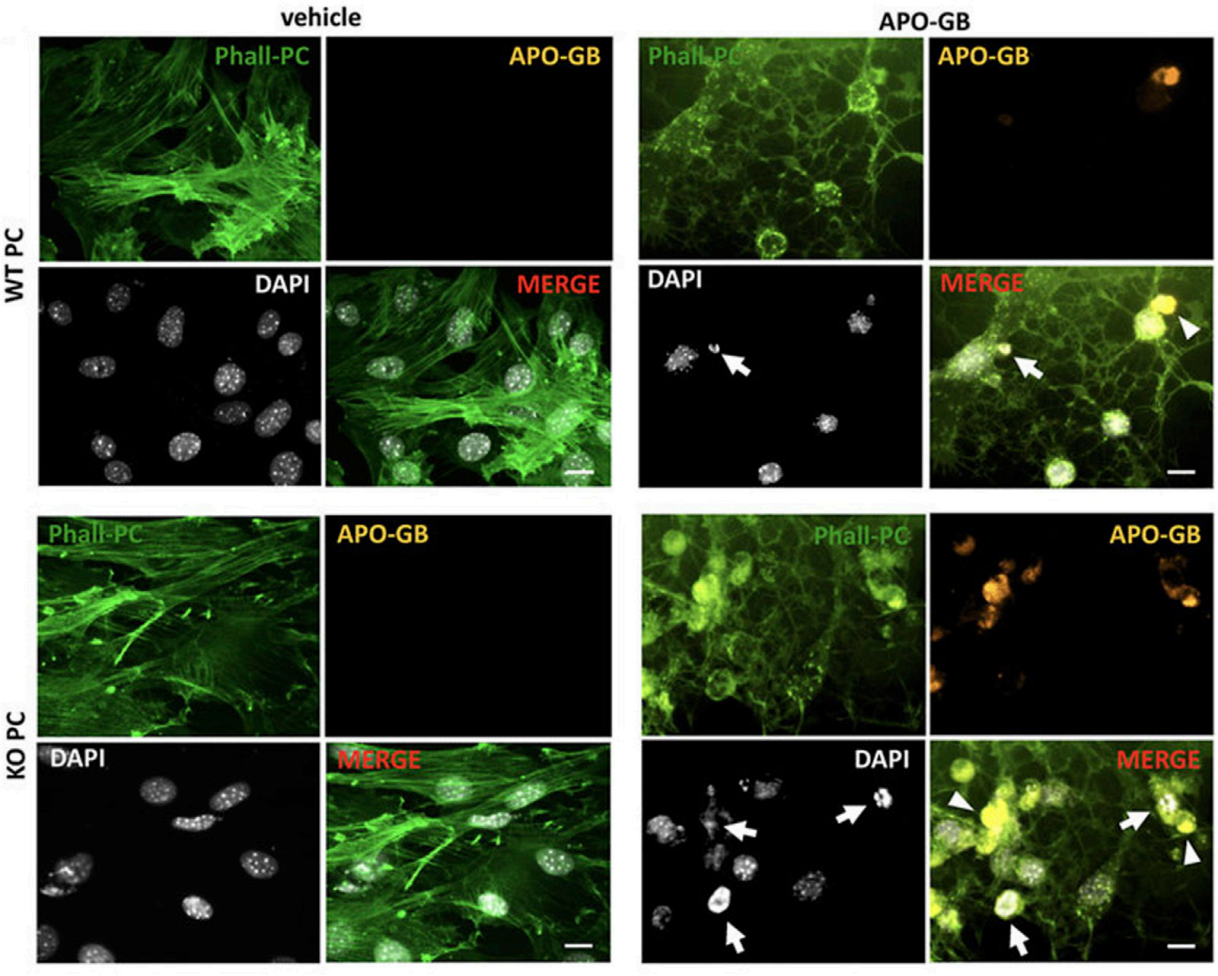
Studying the phagocytic properties of *lamp2a* knock-out pericytes (KO PC) in comparison with wild-type pericytes (WT PC) after incubation with glioblastoma cells. Pericytes were stained with green phalloidin (Phall-PC). These cells can phagocyte pyknotic nuclei (DAPI, white; arrows) and cytoplasmic inclusions (arrowheads) of apoptotic glioblastoma cells [APO-GB stained with DiI and phalloidin (yellow)] in comparison with control wild-type PC without apoptotic glioblastoma cells (vehicle). This assay was done in pentaplicate in U373 and U87 cells. Scale bars: 50 μm. Reproduced with permission (Molina et al., 2022). Copyright 2022, Frontiers in Cell and Developmental Biology.

**FIGURE 7:**
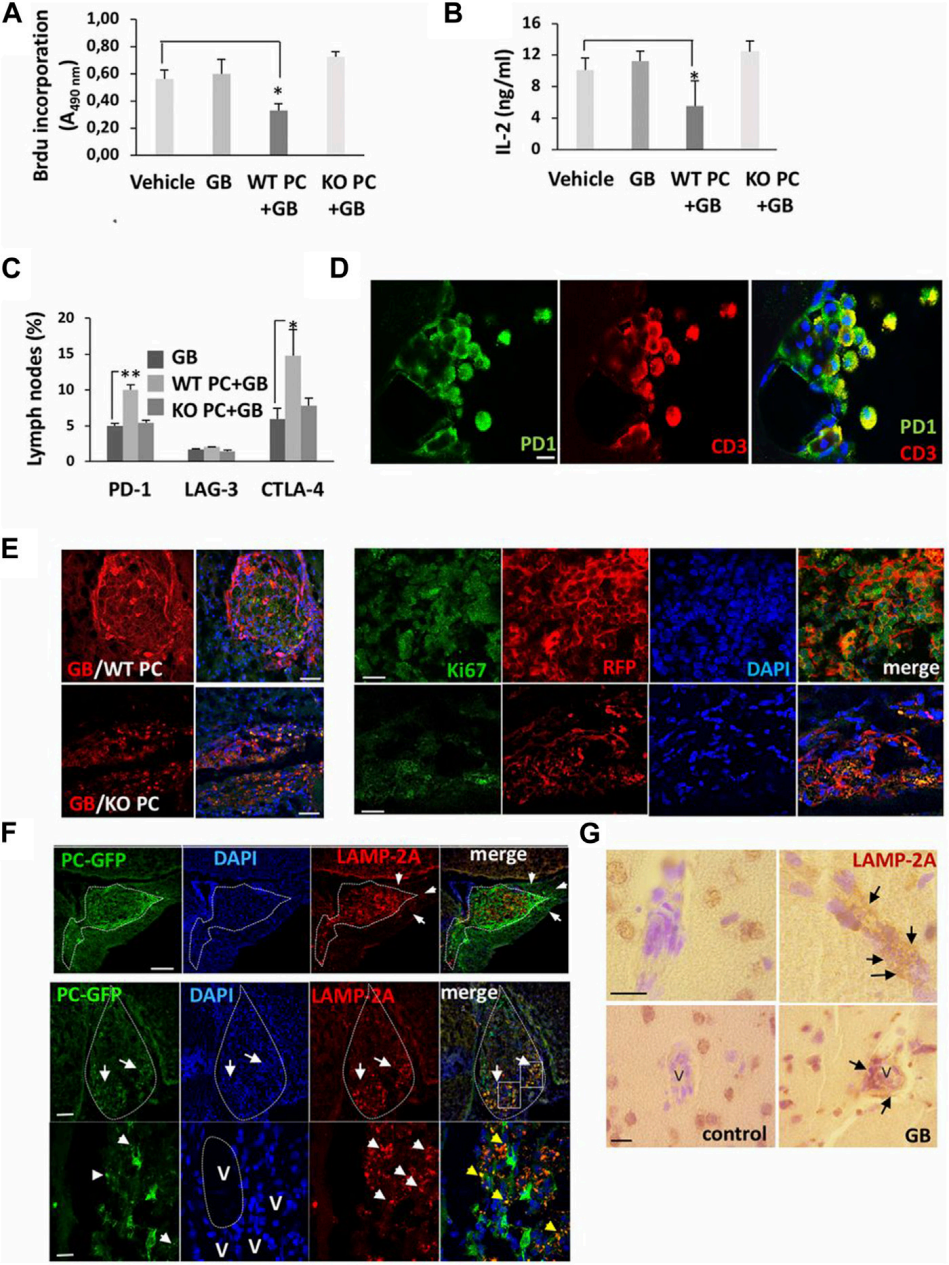
The promotion of CMA in pericytes leads to the development of U87 or U373 glioblastoma cells 4–11 weeks after transplantation. CD4^+^ lymphocytes were isolated from lymph nodes of mice xenografted with wild-type pericytes (WT PC), *lamp2a* knock-out pericytes (KO PC), and RFP + glioblastoma cells (GB), and proliferation capacity **(A)**, IL-2 levels **(B)** and cytotoxicity **(C)** were evaluated and compared to mice xenografted with glioblastoma cells (GB) or PBS (vehicle). Cells were exposed to anti-CD3 and -CD28 antibodies for 72 h *ex vivo* (n = 3; *p < 0.05). Flow cytometry analysis of inhibitory factors in isolated CD4^+^ lymphocytes (*p < 0.05; **p < 0.01) **(C)**. Immunofluorescence images of PD-1 in T lymphocytes isolated from cerebrospinal fluid in mice with xenograft glioblastoma cells and wild-type pericytes (Scale bar: 10 μm) **(D)**. Growth of glioblastoma mass in mice xenografted with glioblastoma cells and wild-type pericytes and compared to the group that received glioblastoma cells with *lamp2a* knock-out pericytes [**(E)**; left panel; Scale bars: 250 μm]. In the right panel **(E)**, numerous red RFP^+^ glioblastoma cells proliferate prominently (Ki-67^+^ cells) in the presence of wild-type pericytes compared to the group juxtaposed to *lamp2a* knock-out pericytes (Scale bar: 50 μm). Measuring LAMP-2A in GFP^+^ pericytes and glioblastoma cells **(F)**. The upper row indicates a strong elevation of LAMP-2A juxtaposed to the perivascular niche (arrows; Scale bar, 100 μm). In the below row, GFP^+^ pericytes are present in the ventral pole of the tumor mass (arrows; Scale bar: 250 μm). Staining of nuclei with DAPI indicates a higher cellularity rate in the ventral pole (arrows). Along with these changes, the number of red LAMP-2A^+^ pericytes at the periphery of tumor mass (arrowheads: LAMP-2A + punctate pattern; blood vessels: V; Scale bar = 45 μm). Immunohistochemistry analysis of LAMP-2A levels (arrows) around the vascular structures (V) in brain samples obtained from the glioblastoma patients **(G)** (Scale bar: 50 μm). (Valdor et al., 2019). Copyright 2019, Proceedings of the National Academy of Sciences of the United States of America.

The interplay between autophagy and other secretory pathways is another approach that can improve cell function and activity. Data indicated shared molecular effectors ATG5, ATG16L1, and Alix between the autophagic system and endosomal system [exosome abscission] (Amini et al., 2021). Exosomes, extracellular vesicles with an average size of 50–200 nm, orchestrate intercellular communication (Dezhakam et al., 2022). It has been indicated that the induction of autophagy is associated with the fusion of autophagosomes with endosomal vesicles, multivesicular bodies (MVBs), to form amphisomes. Along with these changes, certain GTPases like Rab8a and Rab27a are also activated (Villarroya-Beltri et al., 2016). The mutual interaction of the ATG3-ATG12 complex with endosomal Alix can regulate exosome abscission and autophagic response (Amini et al., 2021). Concerning the shared machinery affecting exosome secretion and autophagy, it is hypothesized that the promotion of autophagic response may provoke the endosomal system to alleviate cell injury during pathological conditions. It confirmed that pericytes produced exosomes with the ability to restore the function of ECs via the induction of Claudin-5 and EC‒EC connection in a rat model of spinal cord injury. Meanwhile, the content of HIF-1α, Bax, and aquaporin-4, are reduced at the site of injury via the modulation of the PTEN/AKT signaling axis (Yuan et al., 2019). Given the intricate nature of exosomes and their possible interaction with autophagosomes, direct claims in this regard should be made based on further studies. Of course, the type and intensity of pathological conditions can also alter the content and types of factors released by pericytes into the extracellular matrix (Molina et al., 2022). It has been indicated that the proximity to glioblastoma cells can affect pericyte pro-inflammatory response, oncostatic activity, and cell adhesion capacity in a CMA manner (Molina et al., 2022). The suppression of CMA in *lamp-2a* ^*−/−*^ mice led to an increased intracellular phagosome number and enhanced phagocytic activity (Figure 6) (Molina et al., 2022). Data indicated that administration of exofucosylated deficient *lamp-2a* ^*−/−*^ pericytes into a xenograft mouse model of glioblastoma increased the release of oncostatic factors by pericytes, leading to tumor cell atresia via the recruitment of CD68^+^ macrophages, Iba1^+^ microglia (Molina et al., 2022). It is believed that the activation of pericyte CMA by tumor cells sorts certain cytosolic factors into autophagosomes for lysosomal degradation without affecting pro-oncogenes (Kaushik and Cuervo, 2018; Valdor et al., 2019). In a similar study, CD4^+^ lymphocytes isolated from model mice with wild-type pericytes exhibited reduced activity compared to the glioblastoma mice that received *lamp-2a* ^*−/−*^ pericytes. In glioblastoma mice with knockout *lamp-2a* ^*−/−*^ pericytes, the levels of PD-1 and cytotoxic T lymphocyte-associated protein-4 (CTLA-4) were reduced, indicating the lack of toxicity in immune cells (Figure 7) (Valdor et al., 2019).

## 5 Conclusion

In this review article, the critical role of autophagy was highlighted in vascular cell function, especially pericytes, under physiological and pathological conditions. As expected, autophagy is an early-stage cell resistance mechanism against several insulting conditions such as metabolic disorders. Molecular investigations and histological examination in laboratory scale and animal models uncovered the stimulation of autophagic response in pericytes at early steps following exposure to the insulting conditions. The activation of adaptive autophagy can influence several biological aspects of pericytes within the vessel structure. Autophagy helps the injured pericytes restore their physical connection with the ECs. Under pathological conditions, autophagy can help pericytes generate homotypic and heterotypic TNTs and release exosomes, resulting in the interchange of intracellular organelles and other subcellular components. In contrast to these features, the over-activity of autophagy molecular machinery for a prolonged time can contribute to scavenging system exhaust and provoke the cross-talked cell death pathway like apoptosis in pericytes. Taken together, autophagy can exert its protective effects on pericytes in a time- and intensity-dependent manner. Besides, the crucial role of different autophagy forms should be revisited in the dynamic activity of pericytes and other vascular cells.

## Abbreviations

Advanced glycation end products, AGEs; Alpha-smooth muscle actin, α-SMA; AMP-activated protein kinase, AMPK; Autophagy-related proteins, ATG; Blood-brain-barrier, BBB; Chaperone-mediated autophagy, CMA; c-Jun N-terminal Kinase, JNK; Cytotoxic T lymphocyte-associated protein-4, CTLA-4; Endothelial cells, ECs; Focal adhesion kinase, FAK; G protein signaling 5, RGS-5; Glucose transporter 1, GLUT1; Interleukin-1 beta, IL-1β; Low-density lipoprotein, LDL; Mechanistic target of rapamycin kinase complex 1, mTORC1; Metalloproteinase-2, MMP-2; Microtubule-associated protein 1 light chain 3 alpha, LC3; Multivesicular bodies, MVBs; Neuron-glial antigen 2, NG2; Phosphatase and Tensin homolog, PTEN; Phosphatidylethanolamine, PE; PI3-phosphate, PI3P; Platelet-derived growth factor receptor beta, PDGFR-β; Pre-autophagosomal structures, PAS; Programmed cell death protein 1, PD-1; Reactive oxygen species, ROS; Sigma-1 receptor, σ-1R; Tumor necrosis factor-alpha, TNF-α; Tunneling nanotubes, TNTs; and Vascular endothelial growth factor, VEGF.

## References

[B1] Alarcon-Martinez L. Shiga Y. Villafranca-Baughman D. Belforte N. Quintero H. Dotigny F. (2022). Pericyte dysfunction and loss of interpericyte tunneling nanotubes promote neurovascular deficits in glaucoma. Proc. Natl. Acad. Sci. 119 (7), e2110329119. 10.1073/pnas.2110329119 35135877 PMC8851476

[B2] Alarcon-Martinez L. Villafranca-Baughman D. Quintero H. Kacerovsky J. B. Dotigny F. Murai K. K. (2020). Interpericyte tunnelling nanotubes regulate neurovascular coupling. Nature 585 (7823), 91–95. 10.1038/s41586-020-2589-x 32788726

[B3] Alarcon-Martinez L. Yemisci M. Dalkara T. (2021). Pericyte morphology and function. Histol. Histopathol. 36 (6), 633–643. 10.14670/HH-18-314 33595091

[B4] Alarcon-Martinez L. Yilmaz-Ozcan S. Yemisci M. Schallek J. Kılıç K. Can A. (2018). Capillary pericytes express α-smooth muscle actin, which requires prevention of filamentous-actin depolymerization for detection. Elife 7, e34861. 10.7554/eLife.34861 29561727 PMC5862523

[B5] Álvarez-Arellano L. Pedraza‐Escalona M. Blanco-Ayala T. Camacho-Concha N. Cortés-Mendoza J. Pérez-Martínez L. (2018). Autophagy impairment by caspase-1-dependent inflammation mediates memory loss in response to β-Amyloid peptide accumulation. J. Neurosci. Res. 96 (2), 234–246. 10.1002/jnr.24130 28801921

[B6] Amini H. Rezabakhsh A. Heidarzadeh M. Hassanpour M. Hashemzadeh S. Ghaderi S. (2021). An examination of the putative role of melatonin in exosome biogenesis. Front. Cell Dev. Biol. 9, 686551. 10.3389/fcell.2021.686551 34169078 PMC8219171

[B7] Amirian R. Badrbani M. A. Izadi Z. Samadian H. Bahrami G. Sarvari S. (2023). Targeted protein modification as a paradigm shift in drug discovery. Eur. J. Med. Chem. 260, 115765. 10.1016/j.ejmech.2023.115765 37659194

[B8] Armulik A. Genové G. Mäe M. Nisancioglu M. H. Wallgard E. Niaudet C. (2010). Pericytes regulate the blood–brain barrier. Nature 468 (7323), 557–561. 10.1038/nature09522 20944627

[B9] Asrani K. Murali S. Lam B. Na C. H. Phatak P. Sood A. (2019). mTORC1 feedback to AKT modulates lysosomal biogenesis through MiT/TFE regulation. J. Clin. Invest. 129 (12), 5584–5599. 10.1172/JCI128287 31527310 PMC6877313

[B10] Attwell D. Mishra A. Hall C. N. O'Farrell F. M. Dalkara T. (2016). What is a pericyte? J. Cereb. Blood Flow. Metab. 36 (2), 451–455. 10.1177/0271678X15610340 26661200 PMC4759679

[B11] Barutta F. Bellini S. Kimura S. Hase K. Corbetta B. Corbelli A. (2022). Protective effect of the tunneling nanotube-TNFAIP2/M-sec system on podocyte autophagy in diabetic nephropathy. Autophagy 19, 505–524. 10.1080/15548627.2022.2080382 35659195 PMC9851239

[B12] Bergers G. Benjamin L. E. (2003). Tumorigenesis and the angiogenic switch. Nat. Rev. Cancer 3 (6), 401–410. 10.1038/nrc1093 12778130

[B13] Bergers G. Song S. (2005). The role of pericytes in blood-vessel formation and maintenance. Neuro Oncol. 7 (4), 452–464. 10.1215/S1152851705000232 16212810 PMC1871727

[B14] Berthiaume A. A. Hartmann D. A. Majesky M. W. Bhat N. R. Shih A. Y. (2018). Pericyte structural remodeling in cerebrovascular Health and homeostasis. Front. Aging Neurosci. 10, 210. 10.3389/fnagi.2018.00210 30065645 PMC6057109

[B15] Betsholtz C. (2004). Insight into the physiological functions of PDGF through genetic studies in mice. Cytokine Growth Factor Rev. 15 (4), 215–228. 10.1016/j.cytogfr.2004.03.005 15207813

[B16] Blume Z. I. Lambert J. M. Lovel A. G. Mitchell D. M. (2020). Microglia in the developing retina couple phagocytosis with the progression of apoptosis via P2RY12 signaling. Dev. Dyn. 249 (6), 723–740. 10.1002/dvdy.163 32072708 PMC8714022

[B17] Bondjers C. Kalén M. Hellström M. Scheidl S. J. Abramsson A. Renner O. (2003). Transcription profiling of platelet-derived growth factor-B-deficient mouse embryos identifies RGS5 as a novel marker for pericytes and vascular smooth muscle cells. Am. J. Pathol. 162 (3), 721–729. 10.1016/S0002-9440(10)63868-0 12598306 PMC1868109

[B18] Brown L. S. Foster C. G. Courtney J. M. King N. E. Howells D. W. Sutherland B. A. (2019). Pericytes and neurovascular function in the healthy and diseased brain. Front. Cell Neurosci. 13, 282. 10.3389/fncel.2019.00282 31316352 PMC6611154

[B19] Bujak A. L. Crane J. D. Lally J. S. Ford R. J. Kang S. J. Rebalka I. A. (2015). AMPK activation of muscle autophagy prevents fasting-induced hypoglycemia and myopathy during aging. Cell Metab. 21 (6), 883–890. 10.1016/j.cmet.2015.05.016 26039451 PMC5233441

[B20] Cao W. Li J. Yang K. Cao D. (2021). An overview of autophagy: mechanism, regulation and research progress. Bull. Cancer 108 (3), 304–322. 10.1016/j.bulcan.2020.11.004 33423775

[B21] Chen K. Yong J. Zauner R. Wally V. Whitelock J. Sajinovic M. (2022). Chondroitin sulfate proteoglycan 4 as a marker for aggressive squamous cell carcinoma. Cancers 14 (22), 5564. 10.3390/cancers14225564 36428658 PMC9688099

[B22] Cho H. Kozasa T. Bondjers C. Betsholtz C. Kehrl J. H. (2003). Pericyte-specific expression of Rgs5: implications for PDGF and EDG receptor signaling during vascular maturation. Faseb J. 17 (3), 440–442. 10.1096/fj.02-0340fje 12514120

[B23] de Rooij B. Polak R. Stalpers F. Pieters R. den Boer M. L. (2017). Tunneling nanotubes facilitate autophagosome transfer in the leukemic niche. Leukemia 31 (7), 1651–1654. 10.1038/leu.2017.117 28400620 PMC5508073

[B24] Dezhakam E. Khalilzadeh B. Mahdipour M. Isildak I. Yousefi H. Ahmadi M. (2022). Electrochemical biosensors in exosome analysis; a short journey to the present and future trends in early-stage evaluation of cancers. Biosens. Bioelectron. 222, 114980. 10.1016/j.bios.2022.114980 36521207

[B25] Dias Moura Prazeres P. H. Sena I. F. G. Borges I. d. T. de Azevedo P. O. Andreotti J. P. de Paiva A. E. (2017). Pericytes are heterogeneous in their origin within the same tissue. Dev. Biol. 427 (1), 6–11. 10.1016/j.ydbio.2017.05.001 28479340 PMC6076854

[B26] Di Conza G. Barbaro F. Zini N. Spaletta G. Remaggi G. Elviri L. (2023). Woven bone formation and mineralization by rat mesenchymal stromal cells imply increased expression of the intermediate filament desmin. Front. Endocrinol. 14, 1234569. 10.3389/fendo.2023.1234569 PMC10507407 37732119

[B27] Faulkner A. Lynam E. Purcell R. Jones C. Lopez C. Board M. (2020). Context-dependent regulation of endothelial cell metabolism: differential effects of the PPARβ/δ agonist GW0742 and VEGF-A. Sci. Rep. 10 (1), 7849. 10.1038/s41598-020-63900-0 32398728 PMC7217938

[B28] Filomeni G. Desideri E. Cardaci S. Rotilio G. Ciriolo M. R. (2010). Under the ROS: thiol network is the principal suspect for autophagy commitment. Autophagy 6 (7), 999–1005. 10.4161/auto.6.7.12754 20639698

[B29] Fritzen A. M. Madsen A. B. Kleinert M. Treebak J. T. Lundsgaard A. M. Jensen T. E. (2016). Regulation of autophagy in human skeletal muscle: effects of exercise, exercise training and insulin stimulation. J. Physiol. 594 (3), 745–761. 10.1113/JP271405 26614120 PMC5341711

[B30] Fu D. Yu J. Y. Yang S. Wu M. Hammad S. M. Connell A. R. (2016). Survival or death: a dual role for autophagy in stress-induced pericyte loss in diabetic retinopathy. Diabetologia 59 (10), 2251–2261. 10.1007/s00125-016-4058-5 27475954 PMC5016562

[B31] Gaengel K. Genové G. Armulik A. Betsholtz C. (2009). Endothelial-mural cell signaling in vascular development and angiogenesis. Arterioscler. Thromb. Vasc. Biol. 29 (5), 630–638. 10.1161/ATVBAHA.107.161521 19164813

[B32] Gardiner T. A. Stitt A. W. (2022a). Juxtavascular microglia scavenge dying pericytes and vascular smooth muscle cells in diabetic retinopathy. Int. J. Transl. Med. 2 (1), 41–50. 10.3390/ijtm2010004

[B33] Gardiner T. A. Stitt A. W. (2022b). Pericyte and vascular smooth muscle death in diabetic retinopathy involves autophagy. Int. J. Transl. Med. 2 (1), 26–40. 10.3390/ijtm2010003

[B34] Geranmayeh M. H. Rahbarghazi R. Saeedi N. Farhoudi M. (2022). Metformin-dependent variation of microglia phenotype dictates pericytes maturation under oxygen-glucose deprivation. Tissue Barriers 10(4), 2018928. 10.1080/21688370.2021.2018928 34983297 PMC9620990

[B35] Geranmayeh M. H. Rahbarghazi R. Farhoudi M. (2019a). Targeting pericytes for neurovascular regeneration. Cell Commun. Signal. 17 (1), 26. 10.1186/s12964-019-0340-8 30894190 PMC6425710

[B36] Geranmayeh M. H. Rahbarghazi R. Farhoudi M. (2019b). Targeting pericytes for neurovascular regeneration. Cell Commun. Signal. 17 (1), 26–13. 10.1186/s12964-019-0340-8 30894190 PMC6425710

[B37] Ghanian Z. Mehrvar S. Jamali N. Sheibani N. Ranji M. (2018). Time-lapse microscopy of oxidative stress demonstrates metabolic sensitivity of retinal pericytes under high glucose condition. J. Biophot. 11 (9), e201700289. 10.1002/jbio.201700289 PMC6371775 29577636

[B38] Harrell C. R. Simovic Markovic B. Fellabaum C. Arsenijevic A. Djonov V. Volarevic V. (2018). Molecular mechanisms underlying therapeutic potential of pericytes. J. Biomed. Sci. 25 (1), 21. 10.1186/s12929-018-0423-7 29519245 PMC5844098

[B39] Hassanpour M. Cheraghi O. Rahbarghazi R. Nouri M. (2021). Autophagy stimulation delayed biological aging and decreased cardiac differentiation in rabbit mesenchymal stem cells. J. Cardiovasc. Thorac. Res. 13 (3), 234–240. 10.34172/jcvtr.2021.43 34630972 PMC8493233

[B40] Hassanpour M. Rezaie J. Darabi M. Hiradfar A. Rahbarghazi R. Nouri M. (2020). Autophagy modulation altered differentiation capacity of CD146+ cells toward endothelial cells, pericytes, and cardiomyocytes. Stem Cell Res. Ther. 11 (1), 139–214. 10.1186/s13287-020-01656-0 32216836 PMC7099797

[B41] Hattori Y. (2022). The multiple roles of pericytes in vascular formation and microglial functions in the brain. Life 12 (11), 1835. 10.3390/life12111835 36362989 PMC9699346

[B42] Hayakawa K. Esposito E. Wang X. Terasaki Y. Liu Y. Xing C. (2016). Transfer of mitochondria from astrocytes to neurons after stroke. Nature 535 (7613), 551–555. 10.1038/nature18928 27466127 PMC4968589

[B43] Heidarzadeh M. Gürsoy-Özdemir Y. Kaya M. Eslami Abriz A. Zarebkohan A. Rahbarghazi R. (2021). Exosomal delivery of therapeutic modulators through the blood–brain barrier; promise and pitfalls. Cell and Biosci. 11 (1), 142. 10.1186/s13578-021-00650-0 PMC8296716 34294165

[B44] Hellström M. Kalén M. Lindahl P. Abramsson A. Betsholtz C. (1999). Role of PDGF-B and PDGFR-beta in recruitment of vascular smooth muscle cells and pericytes during embryonic blood vessel formation in the mouse. Development 126 (14), 3047–3055. 10.1242/dev.126.14.3047 10375497

[B45] Herland A. van der Meer A. D. FitzGerald E. A. Park T. E. Sleeboom J. J. F. Ingber D. E. (2016). Distinct Contributions of astrocytes and pericytes to neuroinflammation identified in a 3D human blood-brain barrier on a chip. PLoS One 11 (3), e0150360. 10.1371/journal.pone.0150360 26930059 PMC4773137

[B46] Hussain Y. Singh J. Meena A. Sinha R. A. Luqman S. (2023). Escin‐sorafenib synergy up‐regulates LC3-II and p62 to induce apoptosis in hepatocellular carcinoma cells. Environ. Toxicol. 39, 840–856. 10.1002/tox.23988 37853854

[B47] Ichimiya T. Yamakawa T. Hirano T. Yokoyama Y. Hayashi Y. Hirayama D. (2020). Autophagy and autophagy-related diseases: a review. Int. J. Mol. Sci. 21 (23), 8974. 10.3390/ijms21238974 33255983 PMC7729615

[B48] Jansson D. Rustenhoven J. Feng S. Hurley D. Oldfield R. L. Bergin P. S. (2014). A role for human brain pericytes in neuroinflammation. J. Neuroinflammation 11, 104. 10.1186/1742-2094-11-104 24920309 PMC4105169

[B49] Jiang Q. Gao Y. Wang C. Tao R. Wu Y. Zhan K. (2017). Nitration of TRPM2 as a molecular switch induces autophagy during brain pericyte injury. Antioxidants redox Signal. 27 (16), 1297–1316. 10.1089/ars.2016.6873 28292196

[B50] Julie V. V. T. Hanitriniaina R. Benjamin F. Thierry F. Guylène P. (2022). Autophagy monitoring in cerebral pericytes from alzheimer’s disease mouse model in an inflammatory environment. J. Park. Dis. Alzheimers Dis. 9 (1), 7. 10.13188/2376-922x.1000033

[B51] Kang Y. Li Y. Zhang T. Chi Y. Liu M. (2019). Effects of transcription factor EB on oxidative stress and apoptosis induced by high glucose in podocytes. Int. J. Mol. Med. 44 (2), 447–456. 10.3892/ijmm.2019.4209 31173156 PMC6605469

[B52] Kaushik S. Cuervo A. M. (2018). The coming of age of chaperone-mediated autophagy. Nat. Rev. Mol. Cell Biol. 19 (6), 365–381. 10.1038/s41580-018-0001-6 29626215 PMC6399518

[B53] Kenific C. M. Debnath J. (2016). NBR1-dependent selective autophagy is required for efficient cell-matrix adhesion site disassembly. Autophagy 12 (10), 1958–1959. 10.1080/15548627.2016.1212789 27484104 PMC5079663

[B54] Kim I. Seo J. Lee D. H. Kim Y. H. Kim J. H. Wie M. B. (2023). Ulmus davidiana 60% edible ethanolic extract for prevention of pericyte apoptosis in diabetic retinopathy. Front. Endocrinol. 14, 1138676. 10.3389/fendo.2023.1138676 PMC10206296 37234799

[B55] Kirkin V. McEwan D. G. Novak I. Dikic I. (2009). A role for ubiquitin in selective autophagy. Mol. Cell 34 (3), 259–269. 10.1016/j.molcel.2009.04.026 19450525

[B56] Klionsky D. J. Petroni G. Amaravadi R. K. Baehrecke E. H. Ballabio A. Boya P. (2021). Autophagy in major human diseases. EMBO J. 40 (19), e108863. 10.15252/embj.2021108863 34459017 PMC8488577

[B57] Lee H. W. Xu Y. Zhu X. Jang C. Choi W. Bae H. (2022). Endothelium-derived lactate is required for pericyte function and blood-brain barrier maintenance. Embo J. 41 (9), e109890. 10.15252/embj.2021109890 35243676 PMC9058541

[B58] Lee S. Zeiger A. Maloney J. M. Kotecki M. Van Vliet K. J. Herman I. M. (2010). Pericyte actomyosin-mediated contraction at the cell-material interface can modulate the microvascular niche. J. Phys. Condens Matter 22 (19), 194115. 10.1088/0953-8984/22/19/194115 21386441

[B59] Li C. Guo Z. Liu F. An P. Wang M. Yang D. (2023). PCSK6 attenuates cardiac dysfunction in doxorubicin-induced cardiotoxicity by regulating autophagy. Free Radic. Biol. Med. 203, 114–128. 10.1016/j.freeradbiomed.2023.04.005 37061139

[B60] Li X. Cai Y. Zhang Z. Zhou J. (2022). Glial and vascular cell regulation of the blood-brain barrier in diabetes. Diabetes and Metabolism J. 46 (2), 222–238. 10.4093/dmj.2021.0146 PMC8987684 35299293

[B61] Li X. He S. Ma B. (2020). Autophagy and autophagy-related proteins in cancer. Mol. Cancer 19 (1), 12. 10.1186/s12943-020-1138-4 31969156 PMC6975070

[B62] Lin W.-J. Ma X.-F. Zhou H. R. Xu C. Y. Yu X. Y. Hu Y. X. (2022). Autophagy modulates the migration of retinal pericytes induced by advanced glycation end products. Evidence-Based Complementary Altern. Med. 2022, 2760537. 10.1155/2022/2760537 PMC9771648 36569344

[B63] Liu D. Gao Y. Liu J. Huang Y. Yin J. Feng Y. (2021). Intercellular mitochondrial transfer as a means of tissue revitalization. Signal Transduct. Target. Ther. 6 (1), 65. 10.1038/s41392-020-00440-z 33589598 PMC7884415

[B64] Liu J. Kuang F. Kroemer G. Klionsky D. J. Kang R. Tang D. (2020). Autophagy-dependent ferroptosis: machinery and regulation. Cell Chem. Biol. 27 (4), 420–435. 10.1016/j.chembiol.2020.02.005 32160513 PMC7166192

[B65] Lorzadeh S. Kohan L. Ghavami S. Azarpira N. (2021). Autophagy and the Wnt signaling pathway: a focus on Wnt/β-catenin signaling. Biochimica Biophysica Acta (BBA) - Mol. Cell Res. 1868 (3), 118926. 10.1016/j.bbamcr.2020.118926 33316295

[B66] Losier T. T. Akuma M. McKee-Muir O. C. LeBlond N. D. Suk Y. Alsaadi R. M. (2019). AMPK promotes xenophagy through priming of autophagic kinases upon detection of bacterial outer membrane vesicles. Cell Rep. 26 (8), 2150–2165. 10.1016/j.celrep.2019.01.062 30784596

[B67] Lu G. Du R. Liu Y. Zhang S. Li J. Pei J. (2023). RGS5 as a biomarker of pericytes, involvement in vascular remodeling and pulmonary arterial hypertension. Vasc. Health Risk Manag. 19, 673–688. 10.2147/VHRM.S429535 37881333 PMC10596204

[B68] Ma Q. Zhao Z. Sagare A. P. Wu Y. Wang M. Owens N. C. (2018). Blood-brain barrier-associated pericytes internalize and clear aggregated amyloid-β42 by LRP1-dependent apolipoprotein E isoform-specific mechanism. Mol. Neurodegener. 13 (1), 57. 10.1186/s13024-018-0286-0 30340601 PMC6194676

[B69] Madrakhimov S. B. Yang J. Y. Kim J. H. Han J. W. Park T. K. (2021). mTOR-dependent dysregulation of autophagy contributes to the retinal ganglion cell loss in streptozotocin-induced diabetic retinopathy. Cell Commun. Signal. 19 (1), 29. 10.1186/s12964-020-00698-4 33637094 PMC7913405

[B70] Mailler E. Guardia C. M. Bai X. Jarnik M. Williamson C. D. Li Y. (2021). The autophagy protein ATG9A enables lipid mobilization from lipid droplets. Nat. Commun. 12 (1), 6750. 10.1038/s41467-021-26999-x 34799570 PMC8605025

[B71] Mizushima N. (2010). The role of the Atg1/ULK1 complex in autophagy regulation. Curr. Opin. Cell Biol. 22 (2), 132–139. 10.1016/j.ceb.2009.12.004 20056399

[B72] Molina M. L. García-Bernal D. Salinas M. D. Rubio G. Aparicio P. Moraleda J. M. (2022). Chaperone-mediated autophagy ablation in pericytes reveals new glioblastoma prognostic markers and efficient treatment against tumor progression. Front. Cell Dev. Biol. 10, 797945. 10.3389/fcell.2022.797945 35419364 PMC8997287

[B73] Noda N. N. (2023). Structural view on autophagosome formation. FEBS Lett. 598, 84–106. 10.1002/1873-3468.14742 37758522

[B74] Noda T. Matsunaga K. Taguchi-Atarashi N. Yoshimori T. (2010). Regulation of membrane biogenesis in autophagy via PI3P dynamics. Semin. Cell Dev. Biol. 21 (7), 671–676. 10.1016/j.semcdb.2010.04.002 20403452

[B75] Papinski D. Schuschnig M. Reiter W. Wilhelm L. Barnes C. A. Maiolica A. (2014). Early steps in autophagy depend on direct phosphorylation of Atg9 by the Atg1 kinase. Mol. Cell 53 (3), 471–483. 10.1016/j.molcel.2013.12.011 24440502 PMC3978657

[B76] Pisani F. Castagnola V. Simone L. Loiacono F. Svelto M. Benfenati F. (2022). Role of pericytes in blood–brain barrier preservation during ischemia through tunneling nanotubes. Cell Death Dis. 13 (7), 582. 10.1038/s41419-022-05025-y 35790716 PMC9256725

[B77] Polson H. E. de Lartigue J. Rigden D. J. Reedijk M. Urbé S. Clague M. J. (2010). Mammalian Atg18 (WIPI2) localizes to omegasome-anchored phagophores and positively regulates LC3 lipidation. Autophagy 6 (4), 506–522. 10.4161/auto.6.4.11863 20505359

[B78] Rezabakhsh A. Cheraghi O. Nourazarian A. Hassanpour M. Kazemi M. Ghaderi S. (2017). Type 2 diabetes inhibited human mesenchymal stem cells angiogenic response by over‐activity of the autophagic pathway. J. Cell. Biochem. 118 (6), 1518–1530. 10.1002/jcb.25814 27918077

[B79] Rezabakhsh A. Fathi F. Bagheri H. S. Malekinejad H. Montaseri A. Rahbarghazi R. (2018). Silibinin protects human endothelial cells from high glucose‐induced injury by enhancing autophagic response. J. Cell. Biochem. 119 (10), 8084–8094. 10.1002/jcb.26735 29388698

[B80] Ribatti D. Nico B. Crivellato E. (2011). The role of pericytes in angiogenesis. Int. J. Dev. Biol. 55 (3), 261–268. 10.1387/ijdb.103167dr 21710434

[B81] Ríos J. A. Godoy J. A. Inestrosa N. C. (2018). Wnt3a ligand facilitates autophagy in hippocampal neurons by modulating a novel GSK-3β-AMPK axis. Cell Commun. Signal. 16 (1), 15–12. 10.1186/s12964-018-0227-0 29642895 PMC5896060

[B82] Rowe M. (2020). Investigations of intercellular mitochondrial transfer in neural cells by applied single molecule genotyping.

[B83] Rucker H. K. Wynder H. J. Thomas W. E. (2000). Cellular mechanisms of CNS pericytes. Brain Res. Bull. 51 (5), 363–369. 10.1016/s0361-9230(99)00260-9 10715555

[B84] Rustenhoven J. Jansson D. Smyth L. C. Dragunow M. (2017). Brain pericytes as mediators of neuroinflammation. Trends Pharmacol. Sci. 38 (3), 291–304. 10.1016/j.tips.2016.12.001 28017362

[B85] Salmina A. B. Kharitonova E. V. Gorina Y. V. Teplyashina E. A. Malinovskaya N. A. Khilazheva E. D. (2021). Blood–brain barrier and neurovascular unit *in vitro* models for studying mitochondria-driven molecular mechanisms of neurodegeneration. Int. J. Mol. Sci. 22 (9), 4661. 10.3390/ijms22094661 33925080 PMC8125678

[B86] Schultz N. Byman E. Fex M. Wennström M. (2017). Amylin alters human brain pericyte viability and NG2 expression. J. Cereb. Blood Flow Metabolism 37 (4), 1470–1482. 10.1177/0271678X16657093 PMC5453466 27354094

[B87] Shabkhizan R. Haiaty S. Moslehian M. S. Bazmani A. Sadeghsoltani F. Saghaei Bagheri H. (2023). The beneficial and adverse effects of autophagic response to caloric restriction and fasting. Adv. Nutr. 14 (5), 1211–1225. 10.1016/j.advnut.2023.07.006 37527766 PMC10509423

[B88] Shahabad Z. A. Avci C. B. Bani F. Zarebkohan A. Sadeghizadeh M. Salehi R. (2022). Photothermal effect of albumin-modified gold nanorods diminished neuroblastoma cancer stem cells dynamic growth by modulating autophagy. Sci. Rep. 12 (1), 11774–11818. 10.1038/s41598-022-15660-2 35821262 PMC9276769

[B89] Shi H. Zhang Z. Wang X. Li R. Hou W. Bi W. (2015). Inhibition of autophagy induces IL-1β release from ARPE-19 cells via ROS mediated NLRP3 inflammasome activation under high glucose stress. Biochem. biophysical Res. Commun. 463 (4), 1071–1076. 10.1016/j.bbrc.2015.06.060 26102024

[B90] Shibutani S. T. Yoshimori T. (2014). A current perspective of autophagosome biogenesis. Cell Res. 24 (1), 58–68. 10.1038/cr.2013.159 24296784 PMC3879706

[B91] Shim D. Duan L. Maki C. G. (2021). P53-regulated autophagy and its impact on drug resistance and cell fate. Cancer Drug Resist 4 (1), 85–95. 10.20517/cdr.2020.85 34532654 PMC8443158

[B92] Sil S. Niu F. Tom E. Liao K. Periyasamy P. Buch S. (2019). Cocaine mediated neuroinflammation: role of dysregulated autophagy in pericytes. Mol. Neurobiol. 56 (5), 3576–3590. 10.1007/s12035-018-1325-0 30151726 PMC6393223

[B93] Soriani O. Kourrich S. (2019). The sigma-1 receptor: when adaptive regulation of cell electrical activity contributes to stimulant addiction and cancer. Front. Neurosci. 13, 1186. 10.3389/fnins.2019.01186 31780884 PMC6861184

[B94] Spronk E. Sykes G. Falcione S. Munsterman D. Joy T. Kamtchum-Tatuene J. (2021). Hemorrhagic transformation in ischemic stroke and the role of inflammation. Front. Neurol. 12, 661955. 10.3389/fneur.2021.661955 34054705 PMC8160112

[B95] Stallcup W. B. (2002). The NG2 proteoglycan: past insights and future prospects. J. Neurocytol. 31 (6-7), 423–435. 10.1023/a:1025731428581 14501214

[B96] Stallcup W. B. (2018). The NG2 proteoglycan in pericyte Biology. Pericyte Biology - novel concepts. A. Birbrair. Cham: Springer International Publishing, 5–19. 10.1007/978-3-030-02601-1_2 30523586

[B97] Suzuki K. Kubota Y. Sekito T. Ohsumi Y. (2007). Hierarchy of Atg proteins in pre-autophagosomal structure organization. Genes cells. 12 (2), 209–218. 10.1111/j.1365-2443.2007.01050.x 17295840

[B98] Tasset I. Cuervo A. M. (2016). Role of chaperone-mediated autophagy in metabolism. Febs J. 283 (13), 2403–2413. 10.1111/febs.13677 26854402 PMC4935551

[B99] Thomas W. E. (1999). Brain macrophages: on the role of pericytes and perivascular cells. Brain Res. Brain Res. Rev. 31 (1), 42–57. 10.1016/s0165-0173(99)00024-7 10611494

[B100] Uemura M. T. Maki T. Ihara M. Lee V. M. Y. Trojanowski J. Q. (2020). Brain microvascular pericytes in vascular cognitive impairment and dementia. Front. Aging Neurosci. 12, 80. 10.3389/fnagi.2020.00080 32317958 PMC7171590

[B101] Valdor R. García-Bernal D. Riquelme D. Martinez C. M. Moraleda J. M. Cuervo A. M. (2019). Glioblastoma ablates pericytes antitumor immune function through aberrant up-regulation of chaperone-mediated autophagy. Proc. Natl. Acad. Sci. 116 (41), 20655–20665. 10.1073/pnas.1903542116 31548426 PMC6789971

[B102] van Splunder H. Villacampa P. Martínez-Romero A. Graupera M. (2023). Pericytes in the disease spotlight. Trends Cell Biol. 34, 58–71. 10.1016/j.tcb.2023.06.001 37474376 PMC10777571

[B103] Vicario N. Parenti R. (2022). Connexins signatures of the neurovascular unit and their physio-pathological functions. Int. J. Mol. Sci. 23 (17), 9510. 10.3390/ijms23179510 36076908 PMC9455936

[B104] Villarroya-Beltri C. Baixauli F. Mittelbrunn M. Fernández-Delgado I. Torralba D. Moreno-Gonzalo O. (2016). ISGylation controls exosome secretion by promoting lysosomal degradation of MVB proteins. Nat. Commun. 7 (1), 13588. 10.1038/ncomms13588 27882925 PMC5123068

[B105] Wang J. (2010). Preclinical and clinical research on inflammation after intracerebral hemorrhage. Prog. Neurobiol. 92 (4), 463–477. 10.1016/j.pneurobio.2010.08.001 20713126 PMC2991407

[B106] Wang L. Klionsky D. J. Shen H. M. (2023). The emerging mechanisms and functions of microautophagy. Nat. Rev. Mol. Cell Biol. 24 (3), 186–203. 10.1038/s41580-022-00529-z 36097284

[B107] Weidling I. Swerdlow R. H. (2019). Mitochondrial dysfunction and stress responses in alzheimer’s disease. Biology 8 (2), 39. 10.3390/biology8020039 31083585 PMC6627276

[B108] Wong P. M. Puente C. Ganley I. G. Jiang X. (2013). The ULK1 complex: sensing nutrient signals for autophagy activation. Autophagy 9 (2), 124–137. 10.4161/auto.23323 23295650 PMC3552878

[B109] Xi H. Wang S. Wang B. Hong X. Liu X. Li M. (2022). The role of interaction between autophagy and apoptosis in tumorigenesis (Review). Oncol. Rep. 48 (6), 208. 10.3892/or.2022.8423 36222296 PMC9579747

[B110] Xiang H. Zhou M. Li Y. Zhou L. Wang R. (2023). Drug discovery by targeting the protein‒protein interactions involved in autophagy. Acta Pharm. Sin. B 13, 4373–4390. 10.1016/j.apsb.2023.07.016 37969735 PMC10638514

[B111] Yamamoto H. Kakuta S. Watanabe T. M. Kitamura A. Sekito T. Kondo-Kakuta C. (2012). Atg9 vesicles are an important membrane source during early steps of autophagosome formation. J. Cell Biol. 198 (2), 219–233. 10.1083/jcb.201202061 22826123 PMC3410421

[B112] Yancopoulos G. D. Davis S. Gale N. W. Rudge J. S. Wiegand S. J. Holash J. (2000). Vascular-specific growth factors and blood vessel formation. Nature 407 (6801), 242–248. 10.1038/35025215 11001067

[B113] Yang G. Fan X. Mazhar M. Yang S. Xu H. Dechsupa N. (2022). Mesenchymal stem cell application and its therapeutic mechanisms in intracerebral hemorrhage. Front. Cell Neurosci. 16, 898497. 10.3389/fncel.2022.898497 35769327 PMC9234141

[B114] Yang Z. Wilkie-Grantham R. P. Yanagi T. Shu C. W. Matsuzawa S. I. Reed J. C. (2015). ATG4B (Autophagin-1) phosphorylation modulates autophagy. J. Biol. Chem. 290 (44), 26549–26561. 10.1074/jbc.M115.658088 26378241 PMC4646313

[B115] Ye J. Zhang J. Zhu Y. Wang L. Jiang X. Liu B. (2023). Targeting autophagy and beyond: deconvoluting the complexity of Beclin-1 from biological function to cancer therapy. Acta Pharm. Sin. B 13 (12), 4688–4714. 10.1016/j.apsb.2023.08.008 38045051 PMC10692397

[B116] Ye S. Zhang Y. Wang X. Liang X. Wei M. Zong R. (2021). Autophagy positively regulates Wnt signaling in mice with diabetic retinopathy. Exp. Ther. Med. 22 (4), 1164–1168. 10.3892/etm.2021.10598 34504609 PMC8393590

[B117] Yuan X. Wu Q. Wang P. Jing Y. Yao H. Tang Y. (2019). Exosomes derived from pericytes improve microcirculation and protect blood-spinal cord barrier after spinal cord injury in mice. Front. Neurosci. 13, 319. 10.3389/fnins.2019.00319 31040762 PMC6476953

[B118] Zhang H. Ge S. He K. Zhao X. Wu Y. Shao Y. (2019). FoxO1 inhibits autophagosome-lysosome fusion leading to endothelial autophagic-apoptosis in diabetes. Cardiovasc Res. 115 (14), 2008–2020. 10.1093/cvr/cvz014 30689742

[B119] Zhang Y. Zhang X. Wei Q. Leng S. Li C. Han B. (2020). Activation of sigma-1 receptor enhanced pericyte survival via the interplay between apoptosis and autophagy: implications for blood–brain barrier integrity in stroke. Transl. Stroke Res. 11 (2), 267–287. 10.1007/s12975-019-00711-0 31290080

[B120] Zhao Y. G. Codogno P. Zhang H. (2021). Machinery, regulation and pathophysiological implications of autophagosome maturation. Nat. Rev. Mol. Cell Biol. 22 (11), 733–750. 10.1038/s41580-021-00392-4 34302147 PMC8300085

[B121] Zheng H. J. Zhang X. Guo J. Zhang W. Ai S. Zhang F. (2020). Lysosomal dysfunction-induced autophagic stress in diabetic kidney disease. J. Cell Mol. Med. 24 (15), 8276–8290. 10.1111/jcmm.15301 32583573 PMC7412686

[B122] Zheng Y. Ma H. Yan Y. Ye P. Yu W. Lin S. (2023). Deficiency of filamin A in smooth muscle cells protects against hypoxia-mediated pulmonary hypertension in mice. Int. J. Mol. Med. 51 (3), 22–13. 10.3892/ijmm.2023.5225 36704846 PMC9911089

